# Characterization of the population affiliated to the subsidized health insurance scheme in Colombia: a systematic review and meta-analysis

**DOI:** 10.1186/s12939-022-01818-x

**Published:** 2023-02-07

**Authors:** Laura Mora-Moreo, Kelly Estrada-Orozco, Oscar Espinosa, Lorena Mesa Melgarejo

**Affiliations:** 1 Epidemiology, Evidence Synthesis and Technology Management, Instituto de Evaluación Tecnológica en Salud (IETS), Bogotá, Colombia; 2 Department of Health Services Research and Policy, Faculty of Public Health and Policy, London School of Hygiene & Tropical Medicine, London, England; 3 Directorate of Evidence Synthesis and Management of Health Technologies, Institutode Evaluación Tecnológica en Salud (IETS), Bogotá, D.C. Colombia; 4 Health Technologies and Policies Assessment Group, Instituto de Investigaciones Clínicas, Facultad de Medicina, Universidad Nacional de Colombia, Sede Bogotá, Colombia; 5 Instituto de Investigaciones Clínicas, Facultad de Medicina, Fundación Universitaria Sanitas, Bogotá, Colombia; 6 Directorate of Analytical, Economic and Actuarial Studies at Instituto de Evaluación Tecnológica en Salud (IETS), Bogotá, D.C. Colombia; 7 Economic Models and Quantitative Methods Research Group, Centro de Investigaciones para el Desarrollo, Universidad Nacional de Colombia, Bogotá, Colombia; 8 Directorate of Qualitative Methods and Social Research, Instituto de Evaluación Tecnológica en Salud (IETS), Bogotá, D.C. Colombia

**Keywords:** Social determinants of health, Global health, Public health, Epidemiological factors, Epidemiology

## Abstract

**Background:**

Some reports suggest there are differences in health needs between the population affiliated to the subsidized health insurance scheme (SS) and those affiliated to the contributory health insurance scheme (CS) in Colombia. The objective of this study was to identify the epidemiological profile of the population affiliated to the SS in Colombia and to compare the main epidemiological features of the SS to the CS.

**Methods:**

Following the Preferred Reporting Items for Systematic Reviews and Meta-Analysis (PRISMA) methodology, the search was carried out from 1993, with no other restriction. The information was synthesized into five categories according to the most important risk populations. We estimated combined incidences from epidemiological surveillance data, prevalence ratio, and other measures to estimate the difference between the studied groups. A 95% confidence interval was considered. A random effects model was used weighted by the inverse of the variance of the cumulative incidence calculated for each disease. The risk of bias was assessed using the Joanna Briggs Institute’s critical appraisal tools.

**Results:**

A total of 16,236 articles were identified; 14,972 were excluded after title and abstract screening, 725 articles were verified as full text, and finally 268 articles were included. The relative risk of non-communicable and communicable diseases was lower in the SS than in the CS (RR: 0.37 and 0.72, respectively, *p*-value < 0.05). However, the risk of presenting obstetric and maternal conditions in the SS versus the CS was RR 1.55 for frequent conditions during early childhood, and for other diseases it was RR 1.28 with a *p*-value of < 0.05. The use of health services was different by scheme, with less demand, access, and provision being found in health services in the SS.

**Conclusions:**

This study allowed us to conclude that there are differences in the incidence, prevalence, and use of health services between health affiliation schemes (SS and CS) in Colombia, thereby assisting in decision-making for stakeholders.

**Trial registration:**

PROSPERO Registration number CRD42021279234.

**Supplementary Information:**

The online version contains supplementary material available at 10.1186/s12939-022-01818-x.

## Background

The General System of Social Security in Health (SGSSS) in Colombia is the mechanism by which the public health service is regulated and creates the conditions for the access of the population to all levels of health care. The state integrates this system through the Ministry of Health and Social Protection (MHSP) and other government entities; it began its regulation through Law 100 of 1993, which is the current comprehensive social security system in the country [1]. This law, in order to guarantee access to health care, established that for each affiliated person a Capitation Payment Unit (CPU) is recognized for health-promoting entities (HPEs) [2]. The CPU is defined as the annual value recognized by each person affiliated to the SGSSS to cover the health technologies included in the Health Benefit Plan (HBP). Likewise, Law 100 of 1993 stipulated an insurance system through schemes that include the contributory scheme (CS) and the subsidized scheme (SS), in addition to other so-called “special schemes” (for employees of military forces, public universities, etc.) [3].

All persons linked through a labour contract, public servants, pensioners, and independent workers with the ability to pay can join the CS and, through a monthly contribution to an HPE, they can access health services through health service provider institutions (HSPIs). The people affiliated with this scheme can be dependent or independent workers with income who can guarantee the monthly contributions and, in turn, the additional payments that must be made for health matters, such as moderating fees and co-payments. These payments are proportional to each person’s income [1].

On the other hand, the subsidized scheme is characterized by affiliating people without the ability to pay and those who are vulnerable in the country – that is, those classified as level 1 or 2 in the Beneficiary Selection System (BSS) and priority special populations such as people in forced displacement, the abandoned child population, boys and girls disconnected through armed conflict, indigenous communities, elderly people in protection centres, people from the witness protection programme, street dwellers, and Romani people, among others (according to classification 3 of the BSS). This population can also choose the HPE that provides health-care services through an HSPI [1, 4].

Currently, the value of the financing that is allocated for health coverage in each of the schemes is different, as is provided in Resolution 2503 of 2020, which assigns the budget for the CPU for the year 2021 according to the scheme, either contributory or subsidized. The CPU estimate is the product of the technical-actuarial analysis of the information reported by the HPE to the Ministry of Health, where the main frequencies of use of the health services provided are identified. It is important to note that the quality of the information of the CS is superior to that of the SS. Additionally, government-certified sources are taken into account to consider factors such as demographic changes in the relevant population, the national epidemiological profile, health technologies available in the country, general illnesses and maternal conditions covered in the promotion of health and prevention phases of the disease, and the prevention, diagnosis and treatment of pathologies according to the use of services and levels of care and complexity. Particular characteristics of population subgroups such as the indigenous population are also taken into account, for whom a different percentage of CPU is available due to their socio-cultural, demographic, and epidemiological differences [5].

The analysis of these characteristics to define the monetary values of the CPU for each scheme is carried out in order to collect the differences that may occur in the risk of becoming ill and to present differential health results in the affiliated population. These differences, which are tangible, especially in health outcomes, have been described in the SGSSS in Colombia. In 2015, the Ministry of Health demonstrated the inequities in health in the country in terms of mortality, as well as a possible relationship of this with the population density indices. This analysis also evidenced a positive correlation between infant mortality rates and socio-economic inequity, indicating that the departments that presented improvements in the Gini coefficient maintained lower levels in those rates [6].

Other differences in the consumption of health resources and in the presentation of communicable diseases have also been reported. Mejía et al. [7] published an analytical study that analysed the equity in access to health services in the department of Antioquia, as well as its main determinants, finding that education, age, and type of affiliation to social security were the main factors that determine the access to preventive and curative services. On the other hand, Hilarion-Gaitán et al. [8] evaluated social inequalities in health in Colombia, using the type of affiliation to the health system as a representative parameter of the socio-economic condition. For this, they carried out a descriptive and retrospective study where the incidence rates for events were calculated. In the SS population, 82.31 more cases of Plasmodium falciparum malaria were reported per 100,000 affiliates than those reported in the CS population. Additionally, belonging to the SS was associated with a greater risk of dying from malnutrition in children under 5 years of age, specifically 31.74 times more than for those in the CS.

Although there are some reports and seminal studies that suggest there may be different health needs in the population affiliated to the SS compared to the needs of the CS population in Colombia, determined by differences in the burden of disease, health outcomes, and use of health services, it has not been proven whether these differences exist across the spectrum of diseases, nor is the magnitude of these dissimilarities recognized. Therefore, the objective of this study was to identify the epidemiological profile of the population affiliated to the SS in Colombia, establish if there are differences compared to the CS in terms of incident diseases, prevalent diseases, and use of health services, and estimate the magnitude of differences from statistical analyses of data retrieved in a systematic review of data published in the literature and other sources of information.

## Methods

The protocol for this systematic review (SRL) was registered on PROSPERO (Registration number: CRD42021279234) before starting the literature search. A systematic and exhaustive literature search was carried out. The entire process followed the international quality standards used by the Cochrane Collaboration [9]. The review was guided by the following research question: What is the epidemiological profile and the features of the use of health services for the population affiliated to the SS of Colombia?

We took into account all the population linked to the SS as our target population and the main outcomes assessed were: I) the epidemiological profile of communicable and non-communicable diseases, obstetric and maternal conditions, frequent conditions during early childhood, and other conditions (external causes); and II) the use of health services by the population in each insurance scheme.

### Inclusion and exclusion criteria

The search and selection of studies and other documents was guided by the elements of the structured question. All documents that presented information on i) the population affiliated to the SS in Colombia, ii) data such as incidence, prevalence, the absolute and relative distribution of communicable and non-communicable diseases, other conditions or pathologies, use of health services, and iii) exclusive information on the subsidized scheme or comparative analysis against the CS were included. This SRL was not limited by language or type of study, and the time frame considered was from 1993 to 2021.

### Search strategy

Four bibliographical databases (MEDLINE, Embase, SciELO, and LiLacs) were systematically searched for studies published between January 1st 1993 and September 24th 2021. The threshold was considered to be from 1993 as Law 100 was issued then regarding the classification by health scheme in Colombia.

Relevant literature was identified following search algorithms summarized in Supplementary Table 1. MeSH, Emtree, and DeCS terms such as “epidemiology”, “health insurance”, “subsidized”, “subsidized health insurance scheme”, and “regimens” were used. Additionally, a non-indexed data search was performed on 22 websites related to this topic (Table 1). Studies identified through each database were imported to Mendeley, and then duplicates were identified and removed. The studies were then imported to the program Rayyan [10].

**Table 1:** References found in grey literature and official sources

N°	Databases	Results
1	Ministerio de Salud y Protección Social	663
2	Departamento Administrativo Nacional de Estadistica	4
3	Google Scholar	400
4	Plataforma de Coordinación Interangencial para Refugiados y Migrantes de Venezuela – R4V	88
5	Repositiorio institutional Universidad de Antioquia	241
6	Repositiorio institutional Universidad de Nacional	663
7	Repositiorio institutional Universidad de Pontificia Javeriana	496
8	Repositiorio institutional Universidad de los Andes	1253
9	Repositiorio institutional Universidad del Bosque	62
10	Repositiorio institutional Universidad del Rosario	1423
11	Instituto Nacional de Salud	2010
12	Asociación Colombiana de Empresas de Medicina Integral	48
13	Fundación para la Educación Superior y el Desarrollo	4
14	Fundación para la Investigación y el Desarrollo de la Salud y la Seguridad Social	11
15	Asociación Colombiana de Hospitales	20
16	Contraloría General de la República	400
17	Superintendencia Nacional de Salud	3062
18	Retorno Vital	3
19	Econometría Consultores	2
20	Defensoría del Pueblo	50
21	Cuenta de Alto Costo	5
22	Banco de la República	10
23	Centro de Estudios en Protección Social y Economía de la Salud	29
24	Inter-American Development Bank	33
25	World Bank	21

### Study selection

The title and abstract screening was performed by two reviewers, LMM and KEO. Disagreements were resolved by consensus between the two reviewers using the Rayyan program and a new review of references.

Those studies that met the eligibility criteria previously described were included. The full-text screening was carried out independently by the same two reviewers (LMM and KEO). Disagreements were resolved by consensus between both reviewers. No third reviewer was needed. The complete screening and selection process used was presented using the flow diagram proposed by the PRISMA statement [11].

### Data extraction

The relevant data for this systematic review were extracted by one reviewer (LMM) and independently verified by a second reviewer (KEO), using a data extraction form designed in Excel®, which was adjusted after the pilot extraction carried out with two of the articles. The information was extracted by prioritizing studies with a cohort-type epidemiological design, cases and controls, cross-sectional and descriptive observational ones. Subsequently, the information was extracted from documents and epidemiological surveillance reports, and finally, the documents from repositories of academic databases and grey literature.

### Quality assessment

The quality assessment was conducted by reviewing each study according to the critical appraisal tool made by the Joanna Briggs Institute [12]. These tools assess focus in different aspects, including: (i) type of design; (ii) how outcome variables were assessed; and (iii) if confounding variables were controlled. Quality assessment was performed by one researcher (LMM). Consensus was reached in consultation with a second author (KEO) as needed. The articles assessed were full-text only: event reports, theses, and non-full-text articles were not assessed.

### Analysis of the systematized papers

The synthesis of the information of all the included studies and documents was carried out descriptively. Additionally, synthesis tables were elaborated for the relative frequency measures (incidence and prevalence), or absolute frequency reported in them. The incidences of health conditions were calculated according to five categories of interest: non-communicable diseases, communicable diseases, maternal and obstetric conditions, early childhood health conditions, and other diseases not classified in the previous ones. For each category, the following operational definition was used (Additional file 1: Table 1):

- Non-communicable diseases: They result from a combination of genetic, physiological, environmental, and behavioural factors.
- Communicable diseases: They are contagious or infectious diseases, caused by specific infectious agents or their toxic products in a susceptible host.
- Maternal and obstetric conditions: They are a set of physiological events and pathophysiological alterations that affect women of reproductive age, including in the prenatal, natal, and postnatal periods.
- Common conditions in early childhood: Group of diseases that frequently occur in boys and girls from 0 to 5 years old. In this group of diseases, perinatal conditions and congenital defects are considered.
- Other diseases: This refers to external causes such as accidents, trauma, poisoning, and other events that affect the integrity and health of people that are not related to the health conditions already mentioned.

We presented the information according to each category previously mentioned categorized by affiliation scheme. Additionally, an analysis of “health services use” by scheme was performed.

The incidence estimator was calculated using as denominator the population affiliated to the CS or the SS according to the study period, and as a numerator, the number of cases identified. For example, the human immunodeficiency virus (HIV) report in 2020 identified 5167 cases belonging to the SS and the affiliated population for this scheme in the same year was 24,307,637 people, whilst the number of cases reported in the CS was 6213 out of a total of 21,796,582 affiliated. The number of affiliations by scheme were obtained from the Unique Affiliate Database [13].

On the other hand, combined incidence estimations were generated from data from epidemiological surveillance systems at the national level, using metaregressions considering a 95% confidence interval. In addition, random effects models were used weighted by the inverse of the variance of the cumulative incidence calculated for each pathology and each period of time to the data report, considering the high expected heterogeneity due to: incidence estimators for different health conditions grouped in five categories of analysis (non-communicable diseases, communicable diseases, maternal and obstetric conditions, early childhood health conditions, and other diseases not classified in the above) and different observation periods [14–16]. The heterogeneity values were confirmed by calculating the I^2^ estimate. These results are summarized in “forest plot” graphics. In total, 10 models were built using the statistical software STATA 14 [17].

Frequencies of use of health services were calculated for the people affiliated to each scheme, the SS prevalence ratio between the CS and the odds ratio as a measure of association was reported.

## Results

### Literature search results

In the indexed databases consulted, a total of 5235 references were found and 11,001 references were found in grey literature and official sources such as web pages of official national institutions, repositories of universities, and non-profit organizations. The details of the search results are presented in Table 1.

**Fig. 1:**
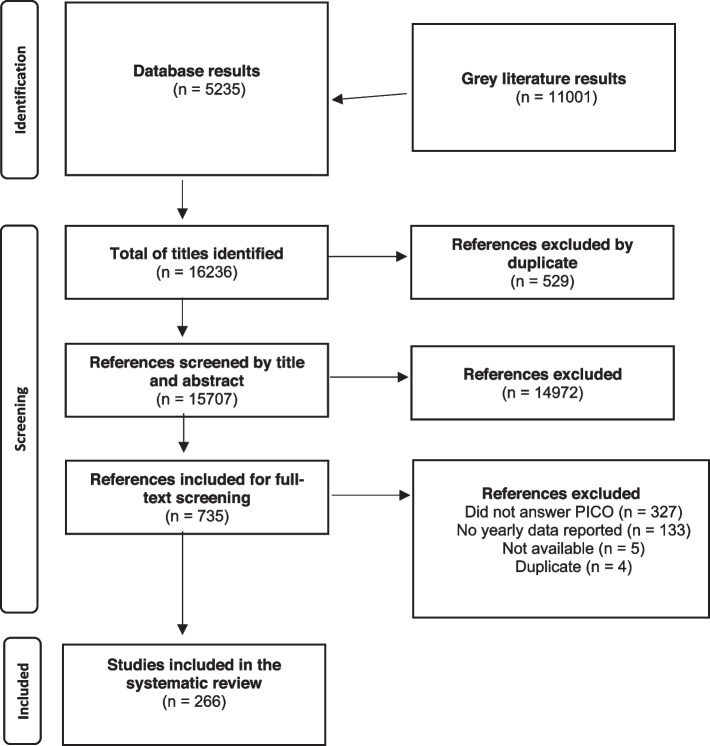
Preferred Reporting Items for Systematic Reviews and Meta-Analyses (PRISMA) diagram

Of the 16,236 references found, 529 were excluded because they were duplicates, 15,707 were screened by title and abstract, and 735 references were evaluated in full text. Finally, 267 documents were included (see Additional file 1: Tables 2 and 3). The information flowchart is detailed in Fig. 1 (PRISMA).

The included studies contain information from 1993 to 2021; 42 articles were obtained from indexed databases [18–59]. Of these, four have a retrospective design [24, 32, 33, 53], two are case series [40, 56], and there is one case-control study [39], five cohort studies [25, 30, 38, 52, 54], 27 prevalence studies (cross-sectional) [18, 20–22, 26, 27, 29, 31, 34–37, 41–49, 51, 55, 57, 58, 60], one descriptive study [19], and two ecological studies [23, 50]. Through Google Scholar, there are 20 references [61–80], 155 annual reports from the Colombian National Institute of Health (INS) [81–228], two reports from R4V [229, 230], one report from the website of the Ministry of Health and Social Protection [231], and two from the “Cuenta de Alto Costo” [232, 233]. The selected references whose sources were the academic repositories of the Colombian universities numbered 44 – 23 from the National University of Colombia [65, 234–255], 11 from the University of Rosario [81, 119, 153, 256–263], three from the University of Antioquia [264–266], three from the University of Andes [267–269], two from the University of Bosque [270, 271], and two from the University Javeriana [272, 273].

Of the documents found, 161 came from public and 105 from private sources. The references that contained information at the national level numbered 192, and there were 74 at the regional, departmental, or municipal level. In regard to the type of document, there were three poster summaries, 64 articles, three report summaries of notification events in PowerPoint format, six technical reports of outbreak studies, 22 infographics, 128 reports of epidemiological surveillance events, two press reports, and 38 academic theses. Among the main cities described in the analysed documents are Bogotá, Barranquilla, Medellín, Cali, Tunja, Cartagena, Chocó, Guainía, Pasto, Popayán, Santander, Villavicencio, Manizales, Bucaramanga, and Pereira.

The number of documents and references according to the category of analysis, i.e. health condition and use of health services, is presented in Table 2.

**Table 2:** Bibliographic references for each category

Category of analysis by health condition or use of health services	Number of documents (%) *n* = 266	References
Non-communicable diseases	58 (21.8)	[18, 20, 26, 30, 32, 35–38, 41, 48, 51, 52, 54, 57, 61, 62, 65, 66, 70, 73, 80, 100–102, 116–118, 130, 132, 133, 137, 146, 147, 149, 150, 154, 194, 234–238, 248, 251, 256, 266, 268, 270, 272, 274–279]
Communicable diseases	128 (48.1)	[8, 32, 33, 35, 37, 39, 40, 50, 52–55, 57, 61–63, 77, 78, 81–84, 89, 92, 94, 96, 97, 99–107, 111–128, 130, 133, 134, 136, 138–141, 145, 147–150, 152–157, 159–162, 164, 173–175, 178, 179, 183, 185, 186, 188, 190, 192, 195–199, 201, 202, 205, 208, 211, 221, 224, 238, 242, 250, 252, 256, 269–284]
Maternal and obstetric conditions	27 (10.1)	[49, 50, 55, 73, 103, 108–110, 145, 151, 158, 169, 170, 198, 218, 225, 231, 243, 247, 248, 285–287]
Common conditions in early childhood	18 (6.7)	[21, 33, 43, 53, 74, 77, 89–92, 109–111, 179, 183, 184, 244, 247]
Other diseases (external causes)	10 (3.7)	[76, 156, 169, 170, 193, 239, 288–291]
Health services	25 (9.3)	[19, 27, 29, 31, 34, 44, 45, 49, 51, 60, 67, 72, 75, 79, 229–231, 240, 252–254, 262, 263, 271, 292]

### General description of the categories of disease in Colombia

#### Non-communicable diseases

Incidence data were collected from reports from the National Institute of Health (INS) for mandatory notification events classified as non-communicable diseases: cancer, rheumatoid arthritis, transplants (kidney, heart, liver, lung), rare diseases, malnutrition, and exposure to fluorine.

The data related to the incidence of the disease comprised 25 reports [100–102, 116–118, 130, 132, 137, 146, 147, 149, 150, 154, 194, 232, 233, 276, 277] and 33 with data on the distribution of diseases in morbid groups of patients [20, 26, 30, 32, 35–38, 41, 48, 52, 54, 61, 62, 65, 66, 70, 73, 235–237, 249, 251, 256, 266, 268, 270, 272, 274, 275, 278, 279, 292].

#### Communicable diseases

INS reports were the main source of information for this category, including data on diseases such as ophidian accidents, attacks by animals transmitting rabies, acute Chagas, chronic Chagas, chikungunya, dengue, chickenpox, malaria, diphtheria, equine encephalitis, meningococcal disease, yellow fever, typhoid and paratyphoid fever, hepatitis B, hepatitis A, infections associated with medical-surgical procedures, acute respiratory infections, leishmaniasis, leprosy, leptospirosis, bacterial meningitis, acute flaccid paralysis, mumps, congenital rubella, measles, congenital syphilis, accidental tetanus, whooping cough, tuberculosis, HIV AIDS, and Zika. Seventy-eight documents were included [9, 21, 29, 31, 35, 36, 46, 49–52, 58, 59, 77–80, 85, 88, 90, 92, 93, 95–99, 101–104, 108–122, 124–127, 130–133, 135–138, 142–147, 149–154, 156–161, 165, 170, 171, 175, 176, 180, 182, 183, 185, 187, 189, 192–196, 198, 199, 202, 205, 206, 208, 218, 221, 262–273] and 15 studies offered information on the distribution of the population in the subsidized scheme [22, 25, 40, 63, 69, 71, 103, 222, 242, 249, 258, 267, 269, 273, 286].

#### Maternal and obstetric conditions

For the conditions that affected women in a fertile period, 10 studies were taken into account that indicated the incidence of the following alterations, namely gestational syphilis, maternal mortality and extreme maternal morbidity [84, 87, 88, 93, 109–111, 113, 114, 145] and six [39, 55, 119, 243, 250, 259] showing the distribution of their population according to the affiliation scheme (CS and SS).

#### Common conditions in early childhood

This category included 11 references, nine from INS reports, and two articles resulting from indexed databases [72, 91, 94, 129, 162, 168, 178, 191, 219, 276, 277], reporting incidences of health conditions and early childhood illness as outcomes such as: underweight at birth, perinatal mortality, late neonatal mortality, and congenital malformations. Five studies showed the distribution of their population for the SS and CS [21, 53, 74, 244, 247].

#### Other diseases

For the category “other diseases”, 10 references were considered, of which eigth events’ data came from INS reports [101, 206, 221, 222, 288–291] and two results from other sources [76, 239]. The incidence of cases of pathologies was taken into account: violence from gender and domestic violence, external causes due to aesthetic procedures and consumption accidents, intoxications, sexual abuse, and maxillofacial trauma.

### Epidemiological behaviour of disease categories according to insurance schemes (SS and CS)

#### Subsidiary scheme

##### Non-communicable diseases

The non-communicable diseases that had the highest incidence during the observation period (2016–2021) was cancer (all types) for the year 2020. Heart, lung, and kidney transplants with living donors represented the fewest incidents. Details of the data can be found in Additional file 2: Table 1.

The combined measure of cumulative incidence for non-communicable diseases in Colombia in the SS during the aforementioned period was 0.000197 (95% CI: 0.000191 to 0.000203). The details of the accumulated incidents by affiliation scheme are presented in Table 3 and their graphic representation in the forest plot of Additional file 2: Illustration 1.

**Table 3:** Cumulative incidence of diseases and other health conditions in Colombia by affiliation scheme during the years 2016–2021

Categories	Subsidiary scheme			Contributive scheme			SS/CS	***p***-value
	Cumulative incidence	95% confidence interval		Cumulative incidence	95% confidence interval		Relative Risk RR	
Non-communicable diseases	0.000197	0.000191	0.000203	0.000526	0.000506	0.000546	0.374000	0.000000
Communicable diseases	0.000124	0.000120	0.000127	0.000171	0.000165	0.000177	0.725146	0.000000
Maternal and obstetric conditions	0.000166	0.000131	0.000201	0.000107	0.000088	0.000127	1.551402	0.000000
Common conditions in early childhood	0.061682	0.060620	0.062744	0.048309	0.047478	0.049141	1.277000	0.000000
Other diseases (external causes)	0.000397	0.000374	0.000420	0.000309	0.000284	0.000333	1.284790	0.000000

*SS* subsidized scheme, *CS* contributory scheme

From the studies that described the frequency distribution of non-communicable diseases according to the affiliation scheme and that were published between 2004 and 2020, information was obtained on different types of cancer, high blood pressure, asthma, cardiovascular diseases, chronic skin diseases, autoimmune joint inflammatory diseases, nutritional deficiencies, and diseases of the oral cavity. The conditions whose number of cases in the SS was higher than those in the CS were breast cancer, chronic diseases in general, and epilepsy. Details are presented in Additional file 3: Table 1.

##### Communicable diseases

For this category, 78 documents were considered including reports and studies. Thirty-two pathologies were included for the observation period 2012 to 2021, with chickenpox and chikungunya having the highest incidence and neonatal tetanus and diphtheria having the lowest incidence of cases (Additional file 2: Table 2). The cumulative incidence for SS was 0.000124 (95% CI: 0.000120 to 0.000127). The details of the cumulative incidences by affiliation scheme are presented in Table 3 and their graphic representation in the forest plot of Additional file 2: Illustration 2.

From the studies that described the frequency distribution of communicable diseases according to the affiliation scheme and that were published between 2011 and 2021, information was obtained on vector-borne diseases, acute respiratory infection, tuberculosis, sexually transmitted infections, and intestinal parasitic infection. Except for acute respiratory infection, multi-drug-resistant tuberculosis, and Zika, all the other communicable conditions had a greater number of cases in the SS than in the CS in the cohorts studied. The conditions with the highest SS/CS ratio were Chagas disease, congenital syphilis, and gestational syphilis. Details are presented in Table 4 [22, 25, 40, 63, 69, 71, 103, 222, 242, 249, 258, 267, 269, 273, 286].

**Table 4:** Distribution of cases by scheme in morbid cohorts of communicable diseases between 2011 and 2021

Author, year	Health condition	Data source	Cases SS	Cases CS	Total	Ratio SS/CS
Curtidor, 2016 [63]	Tuberculosis	TB control follow-up programme	22	203	225	0.1
Urrego, 2019 [269]	Acute respiratory infections	National Demographic and Health Survey	171,051	736,746	915,942	0.2
Nuñez, 2017 [249]	Acute respiratory infections	National Administrative Department of Statistics	23,061	72,493	106,764	0.3
Ruiz, 2017 [267]	Zika	SIVIGILA	126	279	2325	0.5
INS, 2018 [103]	Acute Chagas	INS	9	8	822	1.1
Paniagua, 2021 [25]	Tuberculosis	SIVIGILA	3215	3032	6739	1.1
Angel, 2012 [69]	Sexually transmitted diseases	Profamilia, Hospital of Fontibón, Hospital of Engativá	637	485	1444	1.3
Quintero, 2011 [273]	Sexually transmitted diseases	National Health Survey	12,603	9484	29,760	1.3
Ettenger, 2014 [22]	HIV	National Demographic and Health Survey	3603	2755	10,596	1.3
Gomez, 2019 [242]	HIV	High-Cost Account	14,758	8807	23,803	1.7
Bouwmans, 2016 [258]	Intestinal parasitic infection	Community	121	69	190	1.8
Zabaleta, 2019 [40]	Tuberculosis	National Reference Laboratory	32	17	51	1.9
Agudelo, 2016 [56]	Congenital and gestational syphilis	SIVIGILA	48	10	71	4.8
INS, 2018 [286]	Chronic Chagas	INS	341	33	388	10.3
Echavez, 2018 [71]	Congenital syphilis	SIVIGILA	741	45	845	16.5

*SS* subsidized scheme, *CS* contributory scheme, *HIV* human immunodeficiency virus, *TB* tuberculosis, *INS* National Institute of Health, *SIVIGILA* Public Health Surveillance System

#### Maternal and obstetric conditions

The INS was the main source of information for this category, taking incident data from conditions such as extreme maternal morbidity between the years 2010 and 2021. Of the total number of 10 reports included, extreme maternal morbidity was the condition with the highest incidence, and maternal mortality was the one with the lowest incidence (Additional file 2: Table 3). The pooled incidence measure for the SS was 0.000166 (95% CI: 0.000131 to 0.000201). The details of the accumulated incidences by affiliation scheme are presented in Table 3 and their graphic representation in the forest plot of Additional file 2: Illustration 3.

The studies that described the frequency distribution of obstetric and maternal conditions according to the affiliation scheme were published between 2010 and 2021. It can be seen that the conditions whose number of cases in the SS was higher than those in the CS were maternal mortality and extreme maternal morbidity [39, 55, 119, 243, 250, 259]. Those details are presented in Table 5.

**Table 5:** Distribution of cases by scheme in morbid cohorts of obstetric and maternal conditions, 2010–2021

Author, year	Health condition	Data source	Cases SS	Cases CS	Total	Ratio RS/RC
Salazar, 2017 [243]	Extreme maternal morbidity	SIVIGILA	363	956	1384	0.38
Yepes, 2016 [39]	Extreme maternal morbidity	Second- and third-level institutes	340	485	1011	0.7
Arocha, 2021 [119]	Prenatal care, breastfeeding	Community	536	360	1416	1.49
Álvarez-Sierra, 2020 [55]	Maternal mortality	National Administrative Department of Statistics	28	18	49	1.56
Solarte, 2017 [259]	Extreme maternal mortality	Hospital “la samaritana”	64	15	95	4.27
Soto, 2016 [250]	Maternal mortality	SIVIGILA	15	1	22	13.4
Ayala, 2015 [21]	Prenatal care	Individual Service Provision Registry (ISPR) and vital records	49,300	35,700	85,000	1.4
Catalan, 2010 [53]	Perinatal death	Health-providing institutions	3	1	7	3

*SS* subsidiary scheme, *CS* sontributory scheme, *SIVIGILA* Public Health Surveillance System, *ISPR* Individual Service Provision Registry

#### Common conditions in early childhood

For this category, the information was obtained from nine INS reports and two studies from other sources [72, 91, 94, 129, 162, 168, 178, 191, 219, 276, 277]. Congenital malformations were the pathology with the largest number of new cases. The result of the combined incidence measure was 0.061682 (95% CI: 0.060620 to 0.062744) for the SS. The cumulative incidences by affiliation scheme are presented in Table 3 and their graphic representation in the forest plot of Additional file 2: Illustration 4. Of the studies that described the frequency distribution of non-communicable diseases according to the affiliation scheme and that were published between 2014 and 2016, information was obtained on infant death and congenital malformations, with a ratio of 2.1 to 1 of cases in the SS to the CS [74, 244, 247]. Details are presented in Table 6.

**Table 6:** Distribution of cases by scheme in morbid cohorts of common conditions in early childhood, 2014–2017

Author, year	Health condition	Data source	Cases SS	Cases CS	Total	Ratio RS/RC
Romero, 2017 [244]	Child mortality	Community	30,402	71,852	102,254	0.4
Murica, 2014 [74]	Congenital malformations	Third-level institutions	246	115	416	2.1
Cordoba, 2016 [247]	Child mortality	National Administrative Department of Statistics	396,561	48,265	444,826	8.2

*SS* subsidiary scheme, *CS* contributory scheme

#### Other diseases

This category comprised information from 11 references – nine from the INS between 2018 and 2021, and two studies published in 2015 and 2017 from the literature. For the SS, sexual abuse and intra-family and gender violence were the events with the highest incidence and injuries from external causes caused by accidents related to the consumption of psychoactive substances and aesthetic procedures had the lowest (Additional file 2: Table 5). The cumulative incidence for this scheme was 0.000397 (95% CI: 0.000374 to 0.000420). The details of the accumulated incidents by affiliation scheme are presented in Table 3 and their graphic representation in the forest plot of Additional file 2: Illustration 5.

#### Contributory scheme

##### Non-communicable diseases

For the CS, the non-communicable diseases with the highest incidence were cancer and rheumatoid arthritis, and those with the lowest incidence were kidney and pancreas transplantation and lung transplantation. The cumulative incidence was 0.000526 (95% CI: 0.000506 to 0.000546). The cumulative incidences by affiliation scheme are presented in Table 3 and their graphic representation in the forest plot of Additional file 2: Illustration 6.

From the studies that described the frequency distribution of non-communicable diseases according to the affiliation scheme and that were published between 2004 and 2020, it is evidenced that dental conditions, cancer, and cerebrovascular disease presented a lower number of cases for the CS than for the SS (Additional file 2: Table 1).

##### Communicable diseases

Of the 32 pathologies analysed from 2012 to 2021, the cumulative incidence for the CS was 0.000171 (95% CI: 0.000165 to 0.000177) (Additional file 2: Table 2). Chickenpox, malaria, and dengue were the diseases with the highest incidence, while acute Chagas and equine encephalitis had the lowest incidence. The details of the accumulated incidences by affiliation scheme are presented in Table 3 and their graphic representation in the forest plot of Additional file 2: Illustration 7.

Among the documents that characterized the distribution of pathologies by scheme, it was found that multi-drug-resistant tuberculosis and acute respiratory infections presented a higher ratio of cases to the SS (see Table 4).

#### Obstetric and maternal conditions

For this scheme, the obstetric and maternal condition with the highest incidence was extreme maternal morbidity, while maternal mortality had the lowest. The cumulative incidence was 0.000107 (95% CI: 0.000088 to 0.000127). The details of the accumulated incidences by affiliation scheme are presented in Table 3 and their graphic representation in the forest plot of Additional file 2: Illustration 8.

From the studies that described the frequency distribution of non-communicable diseases according to the affiliation scheme, the ratio of cases of extreme maternal morbidity for the CS is 2.6 to 1 compared to the SS of the SIVIGILA data. For maternal mortality and morbidity and mortality combined, the number of cases is lower in the CS than in the SS [21, 39, 53, 55, 119, 243, 250, 259]. Details are presented in Table 5.

#### Common conditions in early childhood

The early childhood disorder with the highest incidence in the CS was neonatal death, and congenital defects had the lowest incidence. This scheme’s pooled incidence result of common conditions in early childhood was 0.048309 (95% CI: 0.047478 to 0.049141). These data came mostly from INS reports and information was identified between 2009 and 2021. The details of the cumulative incidences by affiliation scheme are presented in Table 3 and their graphic representation in the forest plot of Additional file 2: Illustration 9. Infant mortality presented a ratio of 2.4 for the CS to the SS [39, 45, 69, 217], as identified in Additional file 2: Table 4.

#### Other diseases

In the CS, the cumulative incidence for this category was 0.000309 (95% CI: 0.000284 to 0.000333), with intra-family and gender violence having the highest incidence and alterations due to external causes secondary to cosmetic procedures having the lowest. The source of information was the mandatory reports of the INS SIVIGILA. The details of the cumulative incidences by affiliation scheme are presented in Table 3 and their graphic representation in the forest plot of Additional file 2: Illustration 10.

#### Comparison of disease burden between SS and CS

The relative risk for non-communicable and communicable diseases as categories was lower in the SS than in the CS (RR: 0.37 and 0.72, respectively, *p*-value < 0.05); however, for the other three categories, although differences are found, these represent a greater risk for the population affiliated to the SS, where it is found, for example, that the risk of presenting obstetric and maternal conditions in the SS versus the CS is RR 1.55, while for frequent conditions of early childhood it is RR 1.28, and for other conditions RR 1.28, with a *p*-value of < 0.05 in all cases. Details are presented in Table 3.

#### Non-comparative SS disease burden

This section presents descriptive information from the retrieved articles analysing morbid cohorts with data only for the SS or CS; the study designs do not allow for a formal statistical comparison. The information presented ranges between the years 1993 and 2021, with data related to pathologies such as infant malnutrition, pertussis infection, accidental tetanus, ophidian accident, leishmaniasis, gestational syphilis, maternal mortality, malaria, cancer (solid and haematological tumours), leptospirosis, leprosy, yellow fever, acute Chagas disease, Zika, congenital defects, dengue, puerperal endometritis, infections associated with medical-surgical procedures, chronic diseases, sexually transmitted diseases, maternal complications, HIV, intestinal parasites, perinatal death, and migraine.

Different type of documents: academic theses, reports of mandatory notification events, infographics, technical reports of outbreak studies, scientific articles, and poster summaries were identified, coming from both public and private entities.

##### Non-communicable diseases

The information presented contains data from between 1998 and 2021. The three reports presented by the INS corresponding to the pathology of cancer in children under 18 years of age and acute, moderate, and severe malnutrition in children under 5 years of age for 2021 and 2020, 33, 66, and 64% of cases occurred in the SS, respectively (Table 7).

**Table 7:** Description of studies with information on non-communicable diseases from the SS, 2020–2021

Author, year	Type of document	Nature	Source	Health condition	Total	Cases SS	%
INS, 2021 [86, 91, 111, 114, 126, 131, 132, 134, 138, 159, 163, 171, 185, 192, 199, 210, 217, 276, 288]	Infographic	Public	INS	Cancer in children under 18 years of age	342	113	33.04
INS, 2021 [86, 91, 111, 114, 126, 131, 132, 134, 138, 159, 163, 171, 185, 192, 199, 210, 217, 276, 288]	Infographic	Public	INS	Malnutrition in children under five years of age	8643	5789	66.97
INS, 2020 [84, 89, 106, 109, 116, 124, 130, 133, 136, 166, 174, 180, 187, 188, 194, 197, 201, 206, 208, 211, 215, 221, 281, 284, 287]	Disease report	Public	INS	Malnutrition in children under five years of age	6988	4515	64.61

*INS* National Institute of Health, *SS* subsidized scheme

Agudelo [234] carried out an ecological study published as an academic thesis whose objective was to establish the behaviour of mortality due to child malnutrition in children under 5 years of age in Colombia and its relationship with some social determinants of health; the data source came from the National Administrative Department of Statistics information bases. A total of 12,165 deaths were found and the results show that the correlation between the SS and mortality due to malnutrition in children under 5 years of age, children under 1 year, and children aged between one and four was 0.2057 (Spearman Rho) *p*-value: 0.0000; Rho 0.1933 *p*-value: 0.0000 and 0.2147 (Spearman Rho) *p*-value: 0.0000, respectively.

Afanador-Echeverri et al. [57] presented an abstract in which the current epidemiological situation of migraine in Colombia was analysed, estimating the general prevalence of migraine by geographic area, based on the data obtained from the individual records of provision of services (IRPS) database. This study included a total of 429,816 participants between the years 2014 and 2018, of which 68.8% of the diagnoses were in SS patients and 26.8% in CS patients.

On the other hand, Parra-Lara et al. [18] present a poster summary whose main objective was to analyse the five-year global survival of patients with gastric cancer concerning the health scheme and the place of residence (rural or urban) in a specialized health centre in the city of Cali. A total of 500 cases of gastric cancer were included, and the main outcome (five-year overall survival) was different for patients affiliated to the SS at 14.96% (95% CI: 4.61 to 30.95) versus 26.69% (95% CI: 19.9 to 33.94) for the CS patients (*p*-value: 0.0089).

Castañeda et al. [248] carried out an academic thesis (retrospective cohort) that aimed to describe the burden of disease due to acute paediatric leukaemia (APL) and identify its association with inequalities in terms of affiliation scheme and department of origin in Colombia during the period 2011–2012. The data sources used were the IRPS and vital statistics registered in the DANE of the participating institution. A total of 636 cases of children who died from APL were included. The prevalence of APL for the SS was 9.43 and for the CS it was 9.39, and the mortality rate per 100,000 inhabitants was 1.49 for the CS and 2.19 for the SS.

##### Communicable diseases

The information related to the SS was found in 37 documents, of which 30 came from the INS. These data report information from between 2010 and 2021. Neonatal trachoma and tetanus accounted for all cases in the SS for 2019 and 2020, and neonatal tetanus for 2020 and 2021. The information is detailed in Additional file 3: Table 2.

Cardona et al. (2014) [78], published a study on the prevalence of positive cytology results for bacterial vaginosis, candidiasis, and trichomoniasis between 2010 and 2012 in the city of Medellín. The study aimed to determine the prevalence of positive cytology results for bacterial vaginosis, candidiasis, and vaginal trichomoniasis. The main source of information was obtained from the cervical cancer detection and prevention programme at the Hospital Metrosalud de Medellín in the SS. A total of 206,035 records from the programme databases were included, with the prevalence of bacterial vaginosis being 18%, and that of candidiasis 4.7%, and trichomoniasis 0.8%. On the other hand, Cardona et al. [64] in another study carried out in the city of Medellín between 2010 and 2011, sought to determine the prevalence of bacterial vaginosis in women from Medellín and whose source of information came from 50 health centres and hospital units. It was found that only 41.48% of the women in the SS were users of family planning methods.

Arrivillaga [58] published a cross-sectional study that aimed to evaluate and analyse the association between adherence to treatment and the social position of women living with HIV. This study was carried out between 2006 and 2008 and was part of the project “Social perspective of adherence to treatment in Colombian women with HIV/acquired immunodeficiency syndrome (AIDS)”. A total of 269 women with HIV/AIDS were included in five cities of the country (Cali, Bogotá, Villavicencio, Pasto, and Medellín). The main results showed that the probability of remaining adhered to treatment is three times lower when the patient is affiliated with the SS (OR = 3478, 95% CI: 1957 to 6181) compared to the CS.

Pinzón-Rondón et al. [46], on the other hand, carried out a study aimed at establishing the relationship between intestinal parasitism in children under 6 years of age and living in environmentally protected areas, without an aqueduct service, in El Codito-Bogotá, Colombia. This cross-sectional study included 144 children aged between four and 70 months and demonstrated that, compared with affiliation to the CS, the children affiliated to the SS presented differences in the risk of intestinal parasitic diseases, although they were not statistically significant (OR = 1.49, 95% CI: 0.48 to 4.68).

Borrero et al. [50] published a study that aimed to evaluate the effect of decentralization in the context of the Colombian health system (SGSSS) on the incidence of malaria in Colombian municipalities. This was an ecological study that performed a univariate analysis between the affiliation scheme and the malaria incidence rate between the years 1998 and 2004. The primary source of information was the departmental and municipal health secretaries. A total of 145 cases met the inclusion criteria. In the bivariate analysis between the type of affiliation and the ratio of malaria incidence rates in the municipalities, it was shown that the proportion of the municipal population belonging to the CS was 0.27 (95% CI: 0.11 to 0.64), and the proportion of the municipal population belonging to the SS was 4.23 (95% CI: 1.98 to 9.00). This means that the higher the proportion of the population affiliated to the subsidized regime, the higher the incidence rates of malaria in the municipalities.

#### Maternal and obstetric conditions

Six reports from the INS are listed in Table 8 and show that 56.7% of the cases of gestational syphilis for the year 2021 were presented in the SS; likewise, for the year 2020, there was 54.3% of the same pathology in the SS population.

**Table 8:** Description of articles with information from the SS, 2018–2021

Author, year	Type of document	Nature	Source	Health condition	Total	Cases SS	%
INS, 2021 [86, 91, 111, 114, 126, 131, 132, 134, 138, 159, 163, 171, 185, 192, 199, 210, 217, 276, 288]	Infographic	Public	INS	Gestational syphilis	6391	3624	56.7
INS, 2018 [85, 92, 103, 105, 110, 112, 120, 126, 135, 137, 140, 141, 148, 150, 151, 154, 156, 157, 161, 165, 172, 176, 184, 186, 190, 195, 196, 202, 203, 207, 212, 218, 222, 225, 227, 282, 283]	Disease report	Public	INS	Maternal mortality	523	Maternal mortality ratio is 44.0 cases per 100,000 births	
INS, 2021 [86, 91, 111, 114, 126, 131, 132, 134, 138, 159, 163, 171, 185, 192, 199, 210, 217, 276, 288]	Disease report	Public	INS	Puerperal endometritis	704	286	40.6
INS, 2020 [84, 89, 106, 109, 116, 124, 130, 133, 136, 166, 174, 180, 187, 188, 194, 197, 201, 206, 208, 211, 215, 221, 281, 284, 287]	Disease report	Public	INS	Puerperal endometritis	319	141	44.2
INS, 2020 [84, 89, 106, 109, 116, 124, 130, 133, 136, 166, 174, 180, 187, 188, 194, 197, 201, 206, 208, 211, 215, 221, 281, 284, 287]	Disease report	Public	INS	Puerperal endometritis	704	286	40.6
INS, 2020 [84, 89, 106, 109, 116, 124, 130, 133, 136, 166, 174, 180, 187, 188, 194, 197, 201, 206, 208, 211, 215, 221, 281, 284, 287]	Disease report	Public	INS	Gestational syphilis	5643	3064	54.3

*INS* National Institute of Health, *SS* subsidized scheme

Díaz et al. [264] carried out an ambispective cohort study whose main objective was to determine the factors associated with complications during the third trimester of pregnancy, childbirth, and the puerperium of maternal women belonging to the SS treated in a second-level institution of complexity in Medellín during the year 2012. The cohort included a total of 506 maternal and among the main maternal complications prior to the current pregnancy of the included participants, the prevalence of abortion was 24.5%, pre-eclampsia 8.2%, premature delivery 4.3%, and intrauterine growth retardation 0.9%.

Bolaños et al. [68], in their cross-sectional study, had as their objective to evaluate factors associated with 28 cases of maternal mortality during 2008 in women affiliated to an HPE of the SS, calculating the maternal mortality ratio of 28 deaths per 29,944 live births.

Acevedo [255] carried out a study published as an academic thesis that aimed to identify contextual and individual factors related to maternal health between the years 2011 and 2015 in Vichada. The pregnant women affiliated to the administrative entities of benefit plans (AEBP) of the SS had a maternal mortality ratio of 715.26 deaths per 100,000 live births, and the risk of dying of the pregnant women affiliated to this scheme was five times higher (OR = 5.48; 95% CI: 0.8812 to 226.58) in uninsured pregnant women, although there were no statistically significant differences. There were no cases in women affiliated with the AEBP of the CS or with the exception scheme.

Rivillas et al. [23], in an ecological study whose objective was to measure socio-economic inequality and inequality related to health financing in maternal mortality in Colombia, as well as to identify the potential epicentres of this inequality, indicated that maternal mortality for the indicator of absolute inequity for expenses in health was, in the SS in the year 2012, −10,542, while in 2013 it was −13,979 and in the year 2014 it was −64,781; meanwhile for the CS it was −32,895 in 2012, −23,241 in 2013, and − 17,283 in 2014. This study concluded that there is high inequality in maternal mortality between the regions, and particularly in the SS.

#### Common conditions in early childhood

Cortés et al. [33], who published a retrospective descriptive study that characterized perinatal mortality in Manizales between 2009 and 2012 according to socio-demographic, clinical, and healthcare variables, affiliation scheme, and their relationships, showed that the perinatal mortality rate during the years mentioned was 8.9 per 1000 live births in the CS, and 9.8 per 1000 live births in the SS, while the frequency of perinatal mortality was similar in both affiliation schemes (CS = 50.9% and SS = 49.1%).

#### Behaviour in the use of health-care services according to affiliate schemes in Colombia

The use of health services in affiliation schemes in Colombia is described in studies and reports that informed the distribution of use differently. The number of references included was 21, which reported information between 2009 and 2021. Details are presented in Additional file 3: Table 3.

In this table, the rates of higher frequency of use of health services in the regimens were established, and it is found that the use of radiotherapy, chemotherapy, radiotherapy, and mastectomy, among others, is greater when exposed to the SS compared to the CS (Additional file 3: Table 3).

Additional file 3: Table 4 shows in greater detail a study developed by the MHSP evaluating the quality of care provided in Colombia in 2014. The data showed that there is a greater risk of consumption of services, specifically for drug delivery services, hospitalization, requesting procedures from the HPE in person, and not requesting procedures from the HPE when exposed to the SS than when exposed to the CS.

For the use of services concerning health promotion, prevention, and surgery there were no statistically significant differences between the SS and the CS, and it is notable that the risk of consuming resources for general medicine services, laboratory tests, specialized medicine, emergencies, dentistry, radiography and images, priority appointments, therapies, and requesting medical appointments via telephone or the Internet is lower when you are exposed to the SS compared to the CS.

Zúñiga [271] published an analytical observational study, conducted in 2018, which aimed to analyse inequality in prenatal care live births in Colombia by type of health insurance. The database of the registry of live births was analysed, with a total of 459,238 records, and within the results were found a mean number of prenatal controls for the SS of 6.04 (95% CI: 6.03 to 6.05) while for the CS it was 7.40 (95% CI: 7.39 to 7.41). Likewise, mothers affiliated to the SS were 3.5 times more likely (OR = 3.545; 95% CI: 3.479 to 3.611) to have four or fewer prenatal check-ups than those affiliated to the CS (OR = 0.288; 95% CI: 0.283 to 0.294).

Additional file 3: Table 5, on the other hand, shows the distribution of health services according to the affiliation scheme of the study population. Information was obtained from three studies, carried out between 2009 and 2019, where services such as delivery care and time of health-care attention were evaluated, with a ratio of between 5.41 and 1 cases in the SS to the CS.

#### Quality assessment

Thirty-seven original articles described previously were assessed according to the study design. Other documents included, such as event reports, academic theses, outbreak reports, news, infographics, and epidemiological reports, were not included. Twenty-nine articles were classified as cross-sectional studies. Eight articles [19, 21, 23, 29, 33, 41, 47, 50] were considered of high concern due to the risk of bias mainly because of the lack of information regarding the subject’s settings and the sample size. Three [32, 52, 54] out of seven cohort studies were flagged as being of high concern because of the risk of bias due to unclear information about exposure measures, whether control or exposed groups. One [56] study was evaluated with a case series tool and this was considered to be of low concern in terms of risk of bias. For more information see Additional file 4.

## Discussion

The objective of this study was to identify the epidemiological profile of the population affiliated with the SS in Colombia, to establish whether there are differences compared to the CS in terms of prevalence and incidence of diseases, and use of health services, and to estimate the magnitude of the differences from statistical analyses of the data retrieved in a systematic review of data published in the literature and other sources of information. As a main result, it was determined that there are significant differences in the use of health services, and in the incidence and prevalence of diseases between the population affiliated to the SS and that affiliated to the CS in Colombia. The main differences found account for the existence of a greater burden of disease in terms of morbidity and mortality for the SS in the categories of obstetric and maternal conditions, which include maternal morbidity, extreme maternal morbidity, maternal and perinatal mortality (RR 1.5), the category of frequent childhood conditions with an RR of 1.27, and finally the category of other diseases or conditions, among which injuries due to violent causes stand out (RR 1.28).

These findings can be explained by the greater vulnerability of the population in these categories. The pregnant population, for example, is more vulnerable to access barriers in the provision of health services; these barriers translate into absent or incomplete prenatal controls, which increases the risk of detection, management, and prevention of complications typical of this physiological state with consequences that can be catastrophic for the mother and the product of the pregnancy.

Likewise, it is not unknown that the population affiliated to the SS presents vulnerability characteristics secondary to precarious access to essential public services, the quality of food, transportation, and restricted movement, either due to its location in dispersed areas or to difficult access when found within population centres. These characteristics, added to the existing biological vulnerability in children under 5 years of age, cause the risk of morbidity and mortality to be greater in this age group, while malnutrition, mortality associated with malnutrition, congenital malformations, and postnatal nutritional deficiencies, among others, are problems that occur more frequently in the population affiliated with the subsidized scheme and are mediated beyond the biological substrate by social determinants of health.

Also, the different forms of violence that are monitored in the national information systems were predominant in the population affiliated to the SS. This gender violence, intra-family violence, as well as sexual abuse, intoxication, and consumption accidents, can be understood as a manifestation of unsatisfied basic needs, gender inequalities, a lack of purchasing power, unemployment, inequality in income, level of education [292] and socio-cultural patterns and historical violence such as armed conflict, which demarcate differences in social positions [293].

Other important findings were the significant differences in the incidence of communicable and non-communicable diseases between those affiliated with the SS and CS, with a higher risk of incidence in the population affiliated with the CS. This finding, which is contrary to what is expected given the marked additional risk that has been described for the occurrence of these diseases in a population with a higher degree of vulnerability [55, 294, 295], has two explanations. The first is related to the quality of health information systems and registries at the national level, which constituted a primary source of information for this analysis. It is recognized that the information for the SS and the characterization of the use of health system resources, as well as the burden of disease in its affiliates, is deficient, not representative, and in most cases not available, which may mean in this case an under-reporting of the number of cases and the systematic deviation of these incidents towards an underestimation. Also, structural and intermediate problems of the health system, related to the location of residence of the population affiliated to the SS, deficiencies in transportation, limitations in mobility due to civil security problems, and the location and organization of service-providing institutions, constitute barriers that limit the access of this population to care, so that care is not received in the institutions and consequently they are not recorded in the information systems. This problem also generates a bias in incidence reports with a tendency to underestimate.

The characterization of the prevalence of communicable and non-communicable diseases in the country according to the scheme comes from more representative studies, albeit fragmented by regions or municipalities in many cases. The distribution of cases in the identified morbid cohorts shows a ratio greater than 1 in the cases of patients affiliated to the SS versus the CS who have chronic diseases such as cardiovascular disease and arterial hypertension (ratio 1.3:1), skin lesions, mortality due to nutritional deficiencies, and malnutrition that represent cases of the SS to the CS in a ratio of approximately 2:1, or in the case of epilepsy or other chronic diseases that represent cases of the SS to the CS in a ratio of > 3:1.

This same pattern was observed in communicable diseases such as the occurrence of acute Chagas disease, tuberculosis, sexually transmitted diseases, HIV, intestinal parasitic infection (> 1:1), congenital and gestational syphilis, and chronic Chagas disease (> 4:1). The relative risk of the occurrence of communicable diseases such as leprosy, congenital syphilis, whooping cough, Zika, leishmaniasis, equine encephalitis, malaria, trachoma, and neonatal tetanus is also higher in the population affiliated to the SS than in the CS.

It is especially noteworthy in this study that in the morbid cohorts with cancer in which there is a report in national databases, the largest number of affiliates are found in the CS. Another important finding in this study is related to the presentation of the disease in the population affiliated to the SS, its severity, and the differential behaviour of the clinical course of some of them. In one of the studies included in this analysis, it was evident, at least for the population in Cali, how screening services for malignant breast disease are provided in a 0.5:1 ratio in women from the SS compared to those from the CS, and in women who were diagnosed with breast cancer there was a 2.8 times higher risk of requiring mastectomy among women with this condition affiliated to the SS than in those affiliated to the CS, which may be a proxy for the severity of the disease at the time of diagnosis.

The differences found in the course of the disease are also relevant and induce a reflection on the process of care within the IPS for the population affiliated to one or other scheme. For example, in Bogotá, by 2020, it was found that the risk of having a patient with rheumatoid arthritis with high disease activity at the beginning of the two-year follow-up was lower in the SS than in the CS (OR 0.1); however, upon completing the follow-up time, the risk of presenting high disease activity was higher in the SS than in the CS (OR 1.8), reversing the trend observed at the beginning of follow-up in patients. Also, in delivery care, caesarean sections were more frequent in the CS than in the SS. These findings denote a possible differential approach and other factors that mediate the differential health outcomes in the population that demands and accesses health services in each of these schemes.

Other greater risks that in turn determine a greater burden of disease in SS patients are related to a greater possibility of having failed tuberculosis treatments (OR 1.3) or fewer complete treatments (OR 0.4), and a greater possibility of having refractory epilepsy (OR 4.6), abandonment of breastfeeding (OR 1.3), fewer promotional and preventive activities such as vaginal cytology (OR 0.8), complete vaccination schedules (OR 0.4), or a higher risk of having complete but late vaccination schedules (OR 2.4).

In regard to the use of services offered in the health system, the trend was also evident in terms of relative frequencies towards less use of health services in the SS than in the CS. Such is the case for general medicine services, medication delivery, laboratory tests, specialized medicine, emergency service care, dentistry, radiology and imaging, priority appointment care, and therapies. However, the risk of requiring hospitalization in the population affiliated to the SS is greater than that among those affiliated to the CS (OR 1.65). The use of prevention and promotion health services (P&P) and the performance of surgical procedures do not imply an additional risk in any of the schemes (OR 1.0).

This trend in the differential use of health services, with a lower frequency of use and care in the SS, is understandable to the extent that the population affiliated to this scheme has greater barriers to accessing health services located beyond the provision of services and availability of health technologies. Among them, the characteristics of informality in the work activity generate competition between seeking care and obtaining income that guarantees subsistence. The lower availability of tools that facilitate the request for health-care services and their continuity, such as the telephone or Internet service or the lack of knowledge and appropriate use of this resource, is a fact that again impacts the need for transfers in person to the institutions or service points to request for healthcare services. This confronts them with access barriers generated by problems due to a lack of transportation, mobility, geographical areas and barriers, and the availability of economic resources to access the request for health services. This last phenomenon was also evident in this study, where it was found that the request for health services in person is 3.6 times higher in the SS, so it also occurs with procedures in person (OR 2.1) and in the worst cases the non-request for procedures or services is 1.7 times more frequent in the population affiliated with the SS.

This study, which sought to identify the differences in the incidence and prevalence of diseases and the use of health services between the affiliation schemes (subsidized and contributory) in Colombia, is the first of this nature, and integrates information available in the literature and sources from administrative bases and epidemiological surveillance in Colombia, combining advanced statistical methods for the estimation of combined measures of incidents and analysis of prevalences in each scheme. Additionally, formal statistical tests were carried out, which made it possible to find and quantify the magnitude of the existing differences in the occurrence of disease and the use of health services in Colombia for these two health affiliation schemes.

The sources of information in turn suppose important limitations – among them, the categories of diseases generated a priori could only be fed with information on health conditions and diseases with information available in the literature or the surveillance information bases, however they do not constitute all of the diseases that are part of these categories, and in some cases they are not the most frequently presented.

## Conclusion

This study allowed us to conclude that there are differences in the incidence, prevalence, and use of health services between health affiliation schemes (SS and CS) in Colombia. For the categories configured in the framework of this study as communicable and non-communicable diseases, the data showed a greater risk of becoming ill from this cause in the population affiliated to the CS; however, the certainty in this estimate is very low given the potential biases of information, selection, and measurement detected in the information sources, which even allows an underestimation of the incidences and prevalences of the conditions reported in the SS. For the categories of obstetric and maternal conditions, frequent conditions in early childhood, and other diseases, the risk of occurrence was higher in those affiliated to the SS than among those in the CS. The use of health services is also different by scheme, with less demand, access, and provision in health services for general consultation, dentistry, drug delivery, and emergency consultation, among others, in the SS, probably mediated by social determinants of health, other than the availability of health technologies. However, the risk of requiring hospitalization is greater in those affiliated to the SS.

These results not only allow the differences that are presumed in the configuration of health in the populations belonging to the SS and CS in Colombia to be quantified and quantitatively demonstrated, but they can also guide decision-making regarding the aspects necessary for the actuarial estimation of values ​​to be recognized by people according to the health insurer’s scheme. It is necessary that aspects such as the social characteristics of the population are taken into account in the planning, organization, and management of health risk, given that they are determinants of the burden of disease and health results, as well as the configuration of ways that health care services available are using, as well as barriers that impact the entire care process. There are many limitations in the recovered evidence, so it is necessary to promote studies that seek not only to overcome these limitations and reduce the risk of bias from the design but also to achieve a greater degree of representativeness at the national level.

## Supplementary Information

**Additional file 1: Table 1.** Diagnostic codes. **Table 2.** Search strategy. **Table 3.** References included.

**Additional file 2: Table 1.** Noncommunicable diseases. **Table 2.** Communicable diseases. **Table 3.** Obstetric and maternal conditions. **Table 4.** Frequent conditions in early childhood. **Table 5.** Other diseases. **Illustration 1.** Forest plot of cumulative incidence of noncommunicable diseases in the SS, 2016-2021. **Illustration 2.** Forest Plot of cumulative incidence of communicable disease. **Illustration 3.** Forest plot of cumulative incidence of obstetric and maternal conditions in the SS, 2018-2021. **Illustration 4.** Forest plot of cumulative incidence of common conditions in early childhood in the SS, 2009-2021. **Illustration 5.** Forest plot of cumulative incidence of other diseases in SS, 2015-2021. **Illustration 6.** Forest plot of cumulative incidence of non-communicable diseases in the CS, 2016-2021. **Illustration 7.** Forest plot of cumulative incidence of CS communicable diseases, 2012-2021. **Illustration 8.** Forest plot of cumulative incidence of obstetric and maternal conditions in CS, 2018-2021. **Illustration 9.** Forest plot of cumulative incidence of common conditions in early childhood in the CS, 2009-2021. **Illustration 10.** Forest plot of cumulative incidence of others in CS, 2015-2021.

**Additional file 3: Table 1.** Distribution of cases by regimen in morbid cohorts of non-communicable diseases between 2004 and 2020. **Table 2.** Description of studies with information on communicable diseases from the SS, 2018-2021. **Table 3.** Distribution of cases between regimens. Health services. 2009-2021. **Table 4.** Use of health services. **Table 5.** Distribution of cases by regimen in morbid cohorts in health services. 2009-2019.

**Additional file 4.**

## Data Availability

The data and materials will be made available if required.

## Abbreviations

AIDS: Acquired immunodeficiency syndrome
APL: Acute paediatric leukaemia
AEBP: Administrative entities of benefit plans
BSS: Beneficiary Selection System
CPU: Capitation payment unit
CI: Confidence interval
CS: Contributory scheme
SGSSS: General System of Social Security in Health
HBP: Health benefit plan
HPE: Health-promoting entity
HSPI: Health service provider institution
HIV: Human immunodeficiency virus
IRPS: Individual Service Provision Registry
R4V: Interagency Coordination Platform for Refugees and Migrants from Venezuela
MHSP: Ministry of Health and Social Protection
DANE: National Administrative Department of Statistics
INS: National Institute of Health
OR: Odds ratio
P&P: Promotion of health services
SIVIGILA: Public Health Surveillance System
SS: Subsidized scheme
SRL: Systematic review of literature
TB: Tuberculosis
