# Supplementary material for: Characterization of the population affiliated to the subsidized health insurance scheme in Colombia: a systematic review and meta-analysis
Source: Int J Equity Health. 2023 Feb 7;22:28. doi: 10.1186/s12939-022-01818-x (PMC9903445; doi:10.1186/s12939-022-01818-x)
Supplement: Supplementary file 1 — Additional file 1: Table 1. Diagnostic codes. Table 2. Search strategy. Table 3. References included. [file 12939_2022_1818_MOESM1_ESM.docx]

**Additional file 1.**

Table 1. Diagnostic codes

| **Category** | **ICD 10 codes** |
| --- | --- |
| Communicable diseases | A00-B99, G00-G03, J00-J22, P35.0 |
| Non-communicable diseases | C00-D48, I00-I99, D50-D89, E00-E90, F00-F99, G04-G98, H00-H95, J30-J98, K00-K92, L00-L98, M00-M98, N00-N99, Z00-Z99 |
| Maternal and obstetric conditions | O00-O99 |
| Common conditions in early childhood | P00-P29, P35.1-P96, Q00-Q99 |
| Other diseases | V01-Y89, S00-T98, K35-K46, K56 |

Table 2. Search strategy

| Type or search | New |
| --- | --- |
| Database | MEDLINE/ EMBASE |
| Plataform | PubMed/Ovid |
| Date | 24/09/2021 |
| Time frame | 1993-2020 |
| Other restrictions | None |
| Search strategy | 1. ("epidemiologies"[All Fields] OR "epidemiology"[MeSH Subheading] OR "epidemiology"[All Fields] OR "epidemiology"[MeSH Terms] OR "epidemiology s"[All Fields] OR (("epidemiologies"[All Fields] OR "epidemiology"[MeSH Subheading] OR "epidemiology"[All Fields] OR "epidemiology"[MeSH Terms] OR "epidemiology s"[All Fields]) AND ("profile"[All Fields] OR "profiled"[All Fields] OR "profiler"[All Fields] OR "profilers"[All Fields] OR "profiles"[All Fields] OR "profiling"[All Fields] OR "profilings"[All Fields])) OR ("disease"[MeSH Terms] OR "disease"[All Fields] OR "diseases"[All Fields] OR "disease s"[All Fields] OR "diseased"[All Fields]) OR ("disease"[MeSH Terms] OR "disease"[All Fields] OR "diseases"[All Fields] OR "disease s"[All Fields] OR "diseased"[All Fields]) OR ("clinical protocols"[MeSH Terms] OR ("clinical"[All Fields] AND "protocols"[All Fields]) OR "clinical protocols"[All Fields] OR "regimen"[All Fields] OR "regimens"[All Fields] OR "regimen s"[All Fields])) AND ("epidemiology"[MeSH Subheading] OR "epidemiology"[All Fields] OR "incidence"[All Fields] OR "incidence"[MeSH Terms] OR "incidences"[All Fields] OR "incident"[All Fields] OR "incidents"[All Fields] OR ("epidemiologies"[All Fields] OR "epidemiology"[MeSH Subheading] OR "epidemiology"[All Fields] OR "epidemiology"[MeSH Terms] OR "epidemiology s"[All Fields]) OR ("epidemiology"[MeSH Subheading] OR "epidemiology"[All Fields] OR "incidence"[All Fields] OR "incidence"[MeSH Terms] OR "incidences"[All Fields] OR "incident"[All Fields] OR "incidents"[All Fields]) OR ("epidemiology"[MeSH Subheading] OR "epidemiology"[All Fields] OR "frequency"[All Fields] OR "epidemiology"[MeSH Terms] OR "frequence"[All Fields] OR "frequences"[All Fields] OR "frequencies"[All Fields]) OR ("risk"[MeSH Terms] OR "risk"[All Fields]) OR ("j rehabil assist technol eng"[Journal] OR "rate"[All Fields]) OR ("proportion"[All Fields] OR "proportions"[All Fields]) OR (("cases public health commun mark"[Journal] OR "cases"[All Fields]) AND ("number"[All Fields] OR "numbers"[All Fields])) OR ("epidemiology"[MeSH Subheading] OR "epidemiology"[All Fields] OR "morbidity"[All Fields] OR "morbidity"[MeSH Terms] OR "morbid"[All Fields] OR "morbidities"[All Fields] OR "morbids"[All Fields]) OR ("mortality"[MeSH Terms] OR "mortality"[All Fields] OR "mortalities"[All Fields] OR "mortality"[MeSH Subheading]) OR (("mortality"[MeSH Terms] OR "mortality"[All Fields] OR "mortalities"[All Fields] OR "mortality"[MeSH Subheading]) AND ("risk"[MeSH Terms] OR "risk"[All Fields])) OR ("mortality"[MeSH Terms] OR "mortality"[All Fields] OR ("mortality"[All Fields] AND "rate"[All Fields]) OR "mortality rate"[All Fields]) OR ("fatal"[All Fields] OR "fatalities"[All Fields] OR "fatality"[All Fields] OR "fatally"[All Fields]) OR ("comorbid"[All Fields] OR "comorbidity"[MeSH Terms] OR "comorbidity"[All Fields] OR "comorbidities"[All Fields] OR "comorbids"[All Fields])) AND ("colombia"[MeSH Terms] OR "colombia"[All Fields] OR "colombia s"[All Fields]) AND 1993/01/01:2021/12/31[Date - Publication] AND ("insurance, health"[MeSH Terms] OR ("insurance"[All Fields] AND "health"[All Fields]) OR "health insurance"[All Fields] OR ("health"[All Fields] AND "insurance"[All Fields])) : 170 2. (("epidemiolog*"[Title/Abstract] OR "disease"[Title/Abstract]) AND "colombia"[Title/Abstract]) AND (1993:2021[pdat]): 3358 3. ((Epidemiology or epidemiology profile or disease or diseases or regimens or burden or condition or Morbidity or mortality or mortality risk or mortality rate or fatality or comorbidity or (death or mortality or morbidity or case fatality rate* or case fatality ratio* or fatality rate* or case fatality risk* or severity or severe or lethality or hospitali#ation rate*)) and (health insurance or subsidized or subsidized health insurance scheme) and colombia): 145 4. (health insurance or subsidized or subsidized health insurance scheme).af. AND colombia.af. AND epidemiology.af.: 80 5. ((Epidemiology or epidemiology profile or disease or diseases or regimens or burden or condition or Morbidity or mortality or mortality risk or mortality rate or fatality or comorbidity or (death or mortality or morbidity or case fatality rate* or case fatality ratio* or fatality rate* or case fatality risk* or severity or severe or lethality or hospitali#ation rate*)) and (health insurance or subsidized or subsidized health insurance scheme) and colombia).af.: 465 6. (health insurance or subsidized or subsidized health insurance scheme).af. AND colombia.af.: 677 |
| Identified references | Total MEDLINE via PubMed: 3528  Total MEDLINE via OVID: 225  Total EMBASE: 1142  Total: 4895  Total duplicates: 529  Total evaluated by title and abastract: 4366  Exclusion by title and abstract: 4186  Full text screening: 180  Excluded in full text screening: 138  Included: 42 |

| Type or search | New |
| --- | --- |
| Database | LILACS /Scielo |
| Platform | Website |
| Date | 24/09/2021 |
| Time frame | 1993-2020 |
| Other restrictions | None |
| Search strategy | Scielo:  Subsidiado: 4  LILACS:  Subsidiado: 336 |

**Table 3. References included**

| **N°** | **Title** | **Author, year** | **Type of study** |
| --- | --- | --- | --- |
| 1 | Gender and intrafamily violence in Colombia 2019 | INS, 2019 | Event report |
| 2 | HIV, AIDS and death from AIDS in Colombia 2020 | INS, 2020 | Event report |
| 3 | HIV, AIDS in Colombia 2018 | INS, 2018 | Event report |
| 4 | Report on the behavior of HIV-AIDS Colombia epidemiological period XIII of the year 2016 | INS, 2016 | Event report |
| 5 | Report on the behavior of HIV-AIDS Colombia until the epidemiological period XIII of the year 2015 | INS, 2015 | Event report |
| 6 | HIV-AIDS report up to epidemiological period 13 of 2012 | INS, 2012 | Event report |
| 7 | Mortality due to child malnutrition in children under 5 years of age and its determinants at the municipal level in Colombia 1998 - 2016 | Agudelo, 2019 | Thesis |
| 8 | Situation and response of the migratory phenomenon in health | R4V, 2021 | Infrography |
| 9 | Affiliations to the Health of Venezuelan Refugees and Migrants - Antioquia | R4V, 2021 | Infrography |
| 10 | World Cancer Day 2020 | High Cost Account, 2020 | News |
| 11 | Rheumatoid arthritis, a disease that affects work productivity | High Cost Account, 2020 | News |
| 12 | Varicella event report, Colombia, 2019 | INS, 2019 | Event report |
| 13 | Varicella event report, Colombia, 2018 | INS, 2018 | Event report |
| 14 | Drug-resistant tuberculosis event report, Colombia, 2019 | INS, 2019 | Event report |
| 15 | Drug-resistant tuberculosis event report, Colombia, 2018 | INS, 2018 | Event report |
| 16 | Cancer status in the pediatric population | INS, 2016 | Event report |
| 17 | Tuberculosis event report, Colombia, 2019 | INS, 2019 | Event report |
| 18 | Trachoma event report, Colombia, 2019 | INS, 2019 | Event report |
| 19 | Trachoma event report, Colombia, 2017-2018 | INS, 2018 | Event report |
| 20 | Trachoma Epidemiological period VI. Colombia 2020 | INS, 2020 | Infographic |
| 21 | Whooping cough. Epidemiological period VIII. Colombia 2021 | INS, 2021 | Infographic |
| 22 | Pertussis event report, Colombia, year 2019 | INS, 2019 | Event report |
| 23 | Report of pertussis event, Colombia, year 2018 | INS, 2018 | Event report |
| 24 | Accidental tetanus. Epidemiological period VIII. Colombia 2021 | INS, 2021 | Infographic |
| 25 | Neonatal tetanus event report, Colombia, 2019 | INS, 2019 | Event report |
| 26 | Report of the event accidental tetanos Colombia, 2019 | INS, 2019 | Event report |
| 27 | Neonatal tetanus event report, Colombia, 2020 | INS, 2020 | Event report |
| 28 | Neonatal tetanus event report, Colombia, 2018 | INS, 2018 | Event report |
| 29 | Accidental tetanus event report, Colombia, 2020 | INS, 2020 | Event report |
| 30 | Accidental tetanus event report, Colombia, 2018 | INS, 2018 | Event report |
| 31 | Report of serious events allegedly attributed to vaccination or immunization, Colombia, 2018 | INS, 2018 | Event report |
| 32 | Congenital rubella syndrome, Colombia, 2020 | INS, 2020 | Event report |
| 33 | Congenital rubella syndrome event report, Colombia, 2019 | INS, 2019 | Event report |
| 34 | Low term birth weight event report, Colombia, year 2019 | INS, 2019 | Event report |
| 35 | Low full-term birth weight. Colombia 2018 | INS, 2018 | Event report |
| 36 | Report of attacks and contacts with potentially rabies-transmitting animals - human rabies, colombia, 2019 | INS, 2019 | Event report |
| 37 | Report of ophidian accident event, Colombia, 2019 | INS, 2019 | Event report |
| 38 | Low term birth weight Epidemiological period VIII. Colombia 2021 | INS, 2021 | Infographic |
| 39 | Ophidic accident Epidemiological period VIII. Colombia 2021 | INS, 2021 | Infographic |
| 40 | Report of ophidian accident event, Colombia, 2018 | INS, 2018 | Event report |
| 41 | Chikungunya outbreak in the municipality of San Juan Nepomuceno, Bolivar, Colombia 2014 | INS, 2014 | Outbreak study |
| 42 | Visceral leishmaniasis outbreak in a periurban area in the municipality of Neiva, Huila. Situation from January to July 2017. | INS, 2017 | Outbreak study |
| 43 | Congenital rubella syndrome event report, Colombia, year 2018 | INS, 2018 | Event report |
| 44 | Gestational syphilis and congenital syphilis event report, Colombia, 2019 | INS, 2019 | Event report |
| 45 | Gestational syphilis epidemiological period VIII. Colombia 2021 | INS, 2021 | Infographic |
| 46 | Gestational syphilis and congenital syphilis event report, Colombia, 2020 | INS, 2020 | Event report |
| 47 | Gestational syphilis and congenital syphilis event report, Colombia, 2020 | INS, 2020 | Event report |
| 48 | Gestational syphilis and congenital syphilis report, Colombia, 2018 | INS, 2018 | Event report |
| 49 | Gestational syphilis and congenital syphilis report, Colombia, 2018 | INS, 2018 | Event report |
| 50 | Measles and Rubella Event Report, Colombia, 2019 | INS, 2019 | Event report |
| 51 | Measles and rubella event report, Colombia, 2018 | INS, 2018 | Event report |
| 52 | Measles and rubella event report Colombia, 2018 | INS, 2018 | Event report |
| 53 | Measles and Rubella Event Report, Colombia, 2020 | INS, 2020 | Event report |
| 54 | Mumps event report, Colombia, year 2019 | INS, 2019 | Event report |
| 55 | Mumps event report, Colombia, 2018 | INS, 2018 | Event report |
| 56 | Acute flaccid paralysis event report, colombia, 2020 | INS, 2020 | Event report |
| 57 | Perinatal and late neonatal mortality event report, Colombia, 2018 | INS, 2018 | Event report |
| 58 | Acute flaccid paralysis event report, Colombia, 2018. | INS, 2018 | Event report |
| 59 | Acute flaccid paralysis event report, Colombia, 2019 | INS, 2019 | Event report |
| 60 | Perinatal and late neonatal mortality event report, Colombia, 2019 | INS, 2019 | Event report |
| 61 | Maternal mortality event report, Colombia, 2019 | INS, 2019 | Event report |
| 62 | Extreme maternal morbidity event report, Colombia, period thirteen of 2018 | INS, 2018 | Event report |
| 63 | Maternal mortality event report, Colombia, 2018 | INS, 2018 | Event report |
| 64 | Maternal mortality (early) | INS, 2021 | Infographic |
| 65 | Bacterial meningitis and meningococcal disease event report, Colombia, 2020 | INS, 2020 | Event report |
| 66 | Bacterial meningitis and meningococcal disease event report, Colombia, 2020 | INS, 2020 | Event report |
| 67 | Maternal mortality (early) | INS, 2020 | Infographic |
| 68 | Extreme Maternal Morbidity Report, Colombia, 2019 | INS, 2019 | Event report |
| 69 | Malaria event report, Colombia, 2018 | INS, 2018 | Event report |
| 70 | Bacterial meningitis and meningococcal disease event report, Colombia, 2018 | INS, 2018 | Event report |
| 71 | Bacterial meningitis and meningococcal disease event report, Colombia, 2018 | INS, 2018 | Event report |
| 72 | Cancer in children under  18 years of age | INS, 2021 | Infographic |
| 73 | Diphtheria event report, Colombia 2018 | INS, 2018 | Event report |
| 74 | Dengue event report, Colombia, 2018 | INS, 2018 | Event report |
| 75 | Malaria Epidemiological period VIII. Colombia 2021 | INS, 2021 | Infographic |
| 76 | Mortality associated with congenital anomalies in  Colombia, 1998-2010 | INS, 2010 | Slideshow |
| 77 | Leprosy event report, Colombia, 2019 | INS, 2019 | Event report |
| 78 | Leptospirosis. Epidemiological period VII. Colombia 2021 | INS, 2021 | Infographic |
| 79 | Cutaneous, mucosal and visceral leishmaniasis event report, Colombia, 2019 | INS, 2019 | Event report |
| 80 | Leprosy event report, Colombia 2018 | INS, 2018 | Event report |
| 81 | Leptospirosis event report, Colombia year 2019 | INS, 2019 | Event report |
| 82 | Final report of the leptospirosis event, Colombia, 2015. | INS, 2015 | Event report |
| 83 | Equine encephalitis event report, Colombia, 2018 | INS, 2018 | Event report |
| 84 | Hepatitis a event report, Colombia, 2018 | INS, 2018 | Event report |
| 85 | Behavior of Acute Respiratory Infection Epidemiological weeks 1 to 52 of 2012 | INS, 2012 | Event report |
| 86 | Yellow fever event report, Colombia, 2018 | INS, 2018 | Event report |
| 87 | Chikungunya Colombia event report, 2018 | INS, 2018 | Event report |
| 88 | Typhoid and paratyphoid fever  epidemiological period Colombia 2021 | INS, 2021 | Infographic |
| 89 | Leptospirosis event report, Colombia 2018 | INS, 2018 | Event report |
| 90 | Leprosy epidemiological period vi. Colombia, 2021 | INS, 2021 | Infographic |
| 91 | Acute, moderate and severe malnutrition in children under five years of age  epidemiological period viii. Colombia 2021 | INS, 2021 | Infographic |
| 92 | Yellow fever  epidemiological period vi. Colombia 2021 | INS, 2021 | Infographic |
| 93 | Hepatitis b, c and bd event report, Colombia, 2018. | INS, 2018 | Event report |
| 94 | Mucosal leishmaniasis  Epidemiological period VIII. Colombia 2021 | INS, 2021 | Infographic |
| 95 | Chagas (Acute) Epidemiological period VI. Colombia 2021 | INS, 2021 | Infographic |
| 96 | Diphtheria event report, Colombia, year 2020 | INS, 2020 | Event report |
| 97 | Dengue event report, Colombia, 2019 | INS, 2019 | Event report |
| 98 | ZIKA virus disease Epidemiological period VI. Colombia, 2021 | INS, 2021 | Infographic |
| 99 | Executive Report  Donation and Transplant Network | INS, 2020 | Event report |
| 100 | Congenital defects Epidemiological period VI. Colombia 2021 | INS, 2021 | Infographic |
| 101 | Orphan-rare diseases Colombia, epidemiological period VI, 2021 | INS, 2021 | Infographic |
| 102 | Dengue  Epidemiological period VIII. Colombia 2021 | INS, 2021 | Infographic |
| 103 | Zika and chikungunya virus disease event report, colombia, 2019 | INS, 2019 | Event report |
| 104 | Report on cancer in children under 18 years of age Colombia, 2019 | INS, 2019 | Event report |
| 105 | Executive Report Donation and Transplant Network | INS, 2018 | Event report |
| 106 | Visceral leishmaniasis Epidemiological period VIII. Colombia 2021 | INS, 2021 | Event report |
| 107 | Report of infections associated with medical-surgical procedures in Colombia, 2018 | INS, 2018 | Event report |
| 108 | Report of infections associated with medical-surgical procedures in Colombia, 2018 | INS, 2018 | Event report |
| 109 | Zika virus disease event report, Colombia, 2018 | INS, 2018 | Event report |
| 110 | Congenital syphilis. Epidemiological period VIII. Colombia 2021 | INS, 2021 | Event report |
| 111 | Event report infections associated with medical - surgical procedures, Colombia, 2019 | INS, 2019 | Event report |
| 112 | Acute respiratory infection event report, Colombia, 2020 | INS, 2020 | Event report |
| 113 | Diphtheria event report, Colombia 2019 | INS, 2019 | Event report |
| 114 | Report of moderate and severe acute malnutrition event in children under five years of age, Colombia, 2019 | INS, 2019 | Event report |
| 115 | Congenital defects event report, Colombia year 2018 | INS, 2018 | Event report |
| 116 | Equine encephalitis event report Colombia 2020 | INS, 2020 | Event report |
| 117 | Puerperal endometritis. Epidemiological period XIII. Colombia 2020 | INS, 2021 | Event report |
| 118 | Puerperal endometritis Epidemiological period XIII. Colombia 2020 | INS, 2021 | Event report |
| 119 | Information on kidney transplant recipients with cadaveric donor (DC), Colombia 2016 | INS, 2016 | Power Point presentation |
| 120 | Chikungunya outbreak in the municipality of Mahates, Bolivar, 2014. | INS, 2014 | Report an outbreak |
| 121 | Zika virus disease outbreak in the municipality of Turbaco, Bolívar, Colombia, 2015 | INS, 2015 | Report an outbreak |
| 122 | Acute respiratory infection Colombia 2019 | INS, 2019 | Event report |
| 123 | Moderate and severe acute malnutrition in children under five years Colombia 2018 | INS, 2018 | Event report |
| 124 | Colombia Chagas Disease Event Report, 2018 | INS, 2018 | Event report |
| 125 | Colombia Chagas Disease Event Report, 2018 | INS, 2018 | Event report |
| 126 | External cause injury event report, Colombia 2020 | INS, 2020 | Event report |
| 127 | Malaria outbreak in the municipality of Quibdó Department of Chocó, November 2015 | INS, 2015 | Report an outbreak |
| 128 | Malaria outbreak in the municipalities of Guapi and Timbiquí, Cauca, Colombia, 2014 | INS, 2014 | Report an outbreak |
| 129 | Injuries of external cause. Consumer accidents | INS, 2021 | Event report |
| 130 | Injuries of external cause. Consumer accidents | INS, 2021 | Event report |
| 131 | Chemical poisonings  Colombia 2019 | INS, 2019 | Event report |
| 132 | Poisonings Colombia 2018 | INS, 2018 | Event report |
| 133 | Injuries of external cause. Colombia 2018 | INS, 2018 | Event report |
| 134 | Injuries of external cause. Colombia 2018 | INS, 2018 | Event report |
| 135 | Executive Report Donation and Transplant Network | INS, 2021 | Event report |
| 136 | Annual report on the donation and transplantation network Colombia, year 2019. | INS, 2019 | Event report |
| 137 | Event allegedly attributed to vaccination or immunization - ESAVI (severe) | INS, 2020 | Event report |
| 138 | Confirmed case of acute chagas disease, Rio Iro, Chocó | INS, 2018 | Event report |
| 139 | Zika virus infection  Colombia, 2020 | INS, 2020 | Event report |
| 140 | Events allegedly attributed to vaccination or immunization Colombia, 2020 | INS, 2020 | Event report |
| 141 | Post-vaccination adverse event (severe) | INS, 2021 | Event report |
| 142 | Chikungunya | INS, 2021 | Event report |
| 143 | Puerperal endometritis epidemiological period vi. Colombia 2020 | INS, 2020 | Event report |
| 144 | Puerperal endometritis Epidemiological period VI. Colombia 2020 | INS, 2020 | Event report |
| 145 | Outbreak of an event of unknown etiology in the municipality of El Carmen de Bolívar, Bolívar, 2014 | INS, 2014 | Event report |
| 146 | Congenital defects Epidemiological period VI, Colombia 2020 | INS, 2020 | Event report |
| 147 | Puerperal endometritis Epidemiological period XIII. Colombia 2020 | INS, 2020 | Event report |
| 148 | Gestational syphilis Epidemiological period IX. Colombia 2020 | INS, 2020 | Event report |
| 149 | Unusual increase in malaria cases in Guainía, Colombia 2015 -2017 | INS, 2017 | Event report |
| 150 | Ophidic accident Epidemiological period IX. Colombia 2020 | INS, 2020 | Event report |
| 151 | Dengue Epidemiological period IX. Colombia 2020 | INS, 2020 | Event report |
| 152 | Exposure to fluoride. Colombia 2018 | INS, 2018 | Event report |
| 153 | Leptospirosis Epidemiological period IX. Colombia 2020 | INS, 2020 | Event report |
| 154 | Accidental tetanus epidemiological period IX. Colombia 2020 | INS, 2020 | Event report |
| 155 | Annual report on donation and transplantation network | INS, 2018 | Event report |
| 156 | Annual report. Donation and Transplant Network | INS, 2017 | Event report |
| 157 | Congenital syphilis Epidemiological period IX. Colombia 2020 | INS, 2020 | Event report |
| 158 | Acute, moderate and severe malnutrition in children under five years of age. Epidemiological period IX. Colombia 2020 | INS, 2020 | Event report |
| 159 | Low full-term birth weight. Epidemiological period IX. Colombia 2020 | INS, 2020 | Event report |
| 160 | Orphan-rare diseases Epidemiological period IX. Colombia 2020 | INS, 2020 | Event report |
| 161 | Maternal Mortality (Early) Epidemiological Period VII. Colombia, 2020 | INS, 2020 | Event report |
| 162 | Evaluation study of EPS services by users, in the contributory and subsidized regime | MinSalud, 2014 | Slideshow |
| 163 | Factors associated with maternal mortality in those affiliated to an EPS of the subsidized regime, during 20081 | Amaya, 2008 | Article |
| 164 | Prevalence and factors associated with infection By *C. Trachomatis, N. Gonorrheae*, *t. Vaginalis*, *C. albicans*, syphilis, hiv and bacterial vaginosis in women with symptoms of vaginal infection in three care sites in bogota, colombia, 2010 | Angel, 2012 | Article |
| 165 | High-grade intraepithelial lesions (leiag) in the subsidized, contributory and poor uninsured population in the municipality of Villavicencio, Colombia. 2008 | Baquero, 2010 | Thesis |
| 166 | Association between prostate cancer screening, link to the health system and associated factors in older adults: secondary analysis of the SABE survey Bogotá, Colombia | Borda, 2018 | Article |
| 167 | Mortality of older adults due to nutritional deficiencies in the Departments of Colombia | Cardona, 2012 | Article |
| 168 | Low birth weight: exploration of some risk factors at the san jose de popayan university hospital | Daza, 2009 | Article |
| 169 | Characterization of the population affiliated with the Subsidized Regime in Cali | Rendon, 2001 | Article |
| 170 | Delays in the diagnosis and treatment of women with breast cancer  in Bogotá, Colombia | Pineros, 2011 | Article |
| 171 | Delays in the diagnosis and treatment of women with breast cancer  in Bogotá, Colombia | Pineros, 2011 | Article |
| 172 | Prevalence of positive cytology results for bacterial vaginosis, candidiasis and trichomoniasis in a Social Enterprise of the State of  Medellín-Colombia, 2010-2012. | Cardona, 2014 | Article |
| 173 | Inequities in the diagnosis of major congenital anomalies in newborns in Cali, Colombia. | Murica, 2014 | Article |
| 174 | Use of vaginal cytology in women affiliated  to the subsidized regime treated at the La Manga Hospital Unit in Barranquilla (Colombia), 2006 | Navarro, 2008 | Article |
| 175 | Sexual abuse in women aged 10 to 13 years in the valle del cauca, Colombia | Moran, 2017 | Article |
| 176 | Characterization of people with disabilities in the municipality of Sopó, Cundinamarca, Colombia | Pastran, 2017 | Article |
| 177 | Social determinants of health and quality of life in the adult population of Manizales, Colombia | Garcia, 2017 | Article |
| 178 | Characterization of patients affiliated to the health promoting company of the subsidized regime (eps-s) "emssanar" in the municipalities of pasto and ipiales, Colombia, 2010 | Bravo, 2015 | Article |
| 179 | Prevalence of bacterial vaginosis in users of a health service provider institution in Medellín Colombia | Cardona, 2014 | Article |
| 180 | Factors associated with the proper use of cervical-uterine cytology by women from Cartagena  (Colombia) | Castillo, 2013 | Article |
| 181 | Risk factors for the development of multidrug-resistant tuberculosis in Colombia, 2008 to 2011 | Tanner, 2016 | Article |
| 182 | Epidemiological surveillance of gestational and congenital syphilis  in the department of Córdoba, Colombia, 2012-2016. | Echavez, 2018 | Article |
| 183 | Cancer mortality in Colombia 2001 | Jamramillo, 2004 | Article |
| 184 | Maternal complications in adolescents and adults affiliated with the subsidized regime, 2012 | Diaz, 2015 | Thesis |
| 185 | Social, demographic and morbidity characteristics of patients treated for chronic non-communicable diseases at the ESE Hospital Pedro  Claver Aguirre of the municipality of Toledo-Antioquia 2015-2019. | Perez, 2021 | Thesis |
| 186 | Factors associated with extreme maternal morbidity at the individual level and among the communes and townships of Medellín,  2013-2015 | Salazar, 2017 | Thesis |
| 187 | Access to curative oral health services in schoolchildren in the town of Fontibón de Bogotá DC, 2009. | Echeverria, 2011 | Thesis |
| 188 | Sexually transmitted infections in colombia: analysis based on the 2007 national health survey | Quintero, 2011 | Thesis |
| 189 | Mortality of older adults due to nutritional deficiencies in the Departments of Colombia | Arango, 2012 | Thesis |
| 190 | Impact of subsidized health insurance on access to cervical cytology in Medellín, Colombia | Atehortua, 2014 | Thesis |
| 191 | Characterization of the quality of the care process for colorectal cancer patients treated in 2014 and 2015 at the National Cancer Institute ESE | Torres, 2020 | Thesis |
| 192 | Characterization of mortality in people aged 30 and over due to cardiovascular diseases and its relationship with socio-economic aspects and its link to the general health social security system. Colombia (2005-2013) | Rodriguez, 2017 | Thesis |
| 193 | Aspects related to the number of dental appointments attended by pregnant women in Santander | Barrier, 2017 | Article |
| 194 | Health Insurance and Vaccination Coverage for Children with and without Experience of Forced Displacement in Colombia | Rodriguez, 2008 | Article |
| 195 | Characterization of maxillofacial trauma in the la samaritana hospital in bogota between 2008 and 2013 | Pineda, 2015 | Thesis |
| 196 | Survival of patients with oral squamous cell cancer, treated for the first time, in cancer centers in the period 2000 to 2011, Medellín-Colombia **1** | Posada, 2016 | Article |
| 197 | Factors associated with extreme maternal morbidity at the individual level and among the communes and townships of Medellín,  2013-2015 | Salazar, 2017 | Thesis |
| 198 | Characterization of cerebrovascular accident in Colombia | Duke, 2019 | Report |
| 199 | Characterization of pregnant women with Zika virus in the municipality of Villavicencio, 2016 | Ruiz, 2017 | Article |
| 200 | Social determinants of infant morbidity and mortality due to Acute Respiratory Infection in Bogotá 2015 - 2016 | Urrego, 2019 | Thesis |
| 201 | Economic and health impact of prenatal control in pregnant women affiliated with the subsidized regime in Colombia during 2014 | De la rosa, 2017 | Article |
| 202 | Access to preventive services in the contributory and subsidized health regimes in a stratum two neighborhood of the city of Cali | Grajales, 2011 | Article |
| 203 | Differences in survival due to health insurance in patients with breast cancer treated at a referral cancer center in Medellín, Colombia | Egurrola-Pedraza, 2019 | Article |
| 204 | Inequalities in prescription of hydrochlorothiazide for diabetic hypertensive patients in Colombia | Pinilla, 2011 | Article |
| 205 | Self-report of vaccination in older adults: SABE study Bogotá, Colombia | Cano, 2016 | Article |
| 206 | Health inequalities according to the affiliation regime and events notified to the Surveillance System (Sivigila) in Colombia, 2015 | Hilarion, 2019 | Article |
| 207 | Health insurance for the poor decreases access to HIV testing in antenatal care: evidence of an unintended effect of health insurance reform in  Colombia | Ettenger, 2014 | Article |
| 208 | Does healthcare regime affiliation influence the clinical outcomes of patients with rheumatoid arthritis | Barahona, 2020 | Article |
| 209 | Measuring socioeconomic and health financing inequality in maternal mortality in Colombia: a mixed methods approach | Rivillas, 2020 | Article |
| 210 | Gastric cancer survival and affiliation to health insurance in a middle-income setting | Devries, 2015 | Article |
| 211 | Knowledge, practices and entomological aspects of dengue in Medellín, Colombia: a comparative study  between neighborhoods with high and low incidence | Ortiz, 2018 | Article |
| 212 | Contribution of Health Care Coverage in Cervical Cancer Screening Follow-Up | Garces-Palacios, 2010 | Article |
| 213 | Health care and maternal morbidity and mortality: a hospital-based case-control study in two regions of Colombia (Bogotá and Antioquia), 2009-2011 | Yepes, 2016 | Article |
| 214 | Study and clinical management of minors living with pulmonary tuberculosis patients, Medellín 2010-2011 | Benjumea, 2015 | Article |
| 215 | Social position, gender role, and treatment adherence among Colombian women living with HIV / AIDS: social determinants of health approach | Arrivillaga, 2009 | Article |
| 216 | Prevalence of caries and COP index in 12-year-old school population of the municipality of Copacabana 2013-2014 TT - Caries | Osorio, 2015 | Article |
| 217 | Equity in the use of health services in the General System of Social Security in Health in Colombia | Heads, 2011 | Thesis |
| 218 | Access to drinking water, environmental protection and child intestinal parasitism in El Codito. Bogota Colombia | Finzón-Rondon, 2019 | Article |
| 219 | Characterization of perinatal mortality in manizales, Colombia, 2009-2012 | Cortez, 2014 | Article |
| 220 | Vaccination coverage in Valle del Cauca, 2002 | Cruz, 2003 | Article |
| 221 | Equity in the Detection of Breast Cancer in Colombia | Charry, 2008 | Article |
| 222 | Characterization of congenital and gestational syphilis in Caldas, Colombia | Agudelo, 2016 | Article |
| 223 | Equity in Access to Health Services in Antioquia, Colombia | Mejia, 2007 | Article |
| 224 | Access and satisfaction with curative services: case analysis in affiliated with the contributory regime and affiliated with the subsidized regime in a stratum 2 neighborhood - Cali, Colombia | Grajales, 2015 | Article |
| 225 | Equity in Access to Health Services and Equity in Financing Care in Bogotá | Rubio, 2008 | Article |
| 226 | Delays in maternal mortality in the department of Santander 2012 to 2015 | Álvarez-Sierra, 2020 | Article |
| 227 | Current situation of migraine in Colombia: analysis based on the health services information system (RIPS) | Afanador Echeverri, 2019 | Abstract |
| 228 | Health disparities and gastric cancer survival in Cali, Colombia: A hospital-based study | Parra-Lara, 2019 | Abstract |
| 229 | Evaluation of emotional, social and access  needs of patients diagnosed with chronic  myeloid leukemia in Colombia. | Rocha, 2019 | Abstract |
| 230 | Decentralization and reform: what is its impact on the incidence of malaria in Colombian municipalities? | Borrero, 2012 | Article |
| 231 | Access, security and comprehensiveness in the registries of Colombian pregnant women between 2007 and 2009 | Ayala, 2015 | Article |
| 232 | Characterization of the patients taken to cesarean section according to the Robson model and exploration of associated factors in pregnant women treated at the San José University Hospital in the city of  Popayán-Colombia. January 1, 2016 to June 30, 2016. | Anaya, 2017 | Article |
| 233 | Accessibility factors related to perinatal death | Catalan, 2010 | Article |
| 234 | Determinants of the use of different levels of care in the General System of Social Security in Health  and the Unified Health System in Colombia and Brazil | Garcia-Subirats, 2014 | Article |
| 235 | Equity in access to treatment for breast cancer in Colombia | Charry, 2009 | Article |
| 236 | Mortality and morbidity trends due to  pertussis in Colombia, 2002-2012 | Cardona, 2016 | Article |
| 237 | Association between delay in diagnosis and advanced clinical stage of breast cancer at the time  of consultation in four cancer centers in Medellín, Colombia, 2017. Cross-sectional study | Martinez perez, 2019 | Article |
| 238 | Case series: extremely drug-resistant tuberculosis  in Colombia, 2006-2016 | Zabaleta, 2019 | Article |
| 239 | Prevalence of skin lesions associated with physical dependence in nursing homes and health institutions in Tunja | Hernandez, 2019 | Article |
| 240 | Health insurance coverage, neonatal mortality and caesarean section deliveries: an analysis of vital registration data in Colombia | Houweling, 2016 | Article |
| 241 | Factors Associated with Unsuccessful Outcomes of Tuberculosis Treatment in 125 Municipalities in Colombia 2014 to 2016 | Paniagua, 2021 | Article |
| 242 | Incidence and demographic determinants of acute lymphoid leukemia in patients with pediatric cancer, Antioquia | Gomez, 2020 | Article |
| 243 | Inequalities in health insurance in prenatal care in Colombia Insurance health disparities in antenatal care in Colombia | Zuñiga, 2020 | Thesis |
| 244 | Descriptive Analysis of Morbidity and Mortality Due to Acute Lymphoid Leukemia in Children Under 20 Years of Age According to Department and Insurance Regime in Colombia 2010-2015 | Garcia, 2020 | Thesis |
| 245 | Association between sociodemographic conditions and uncontrolled epilepsy in populations with limited access to neurology services | Soto, 2019 | Thesis |
| 246 | Barriers in access to healthcare in countries with different health systems. A cross-sectional study in municipalities of central Colombia and north-eastern Brazil | Garcia-Subirats, 2014 | Article |
| 247 | Prevalence of intestinal parasites in preschool children from vulnerable neighborhoods of Bogotá | Bouwmans, 2016 | Article |
| 248 | Associated factors of multidimensional poverty with taking cytology in Colombia. | Sanchez Rincon, 2020 | Thesis |
| 249 | Characteristics of the caregivers associated with the dental consultation of Colombian children under 5 years of age. | Barrier, 2020 | Thesis |
| 250 | Evaluation of the time free from asthma attacks in children aged 4 to 11 years in relation to social factors. Pilot study in Bogota | Novoa, 2019 | Thesis |
| 251 | Characterization and profiles of patients with extreme maternal morbidity, intensive care unit, hospital la samaritana, 2008 -2014 | Solarte, 2017 | Thesis |
| 252 | Economic, social and health determinants that affect exclusive breastfeeding in Colombia for the year 2010 | Arocha, 2021 | Thesis |
| 253 | Sexual and reproductive health: aspects of analysis for the department of casanare | Orduz, 2016 | Thesis |
| 254 | Socio-economic determinants of global child malnutrition in the town of ciudad bolívar (bogota, colombia) in 2011 | Ducuara, 2012 | Thesis |
| 255 | Social determinants and their association with not taking cervical vaginal cytology in the rural population of Colombia. | Sosa, 2020 | Thesis |
| 256 | Comparison of two contraceptive counseling models for HIV positive women from a health equity perspective Colombia 2015-2017 | Gomez, 2019 | Thesis |
| 257 | Sociodemographic characterization of maternal health in Vichada, 2011-2915: a view from morbidity and mortality | Acevedo, 2019 | Thesis |
| 258 | Factors associated with the inequality of Infant Mortality in the towns of Bogotá, 2013 | Romero, 2017 | Thesis |
| 259 | Nursing intervention for self-management in adults with colorectal cancer undergoing surgical treatment | Vergara, 2020 | Thesis |
| 260 | Social Determinants and Mortality in Children under 5 Years of Age in the Department of Chocó | Cordoba, 2016 | Thesis |
| 261 | Inequality in burden of disease due to acute respiratory infection (ARI) and acute diarrheal disease (ADD) under 5 years of age, Colombia 2010. | Calderon, 2016 | Thesis |
| 262 | Association of the burden of disease due to acute pediatric leukemia and health inequalities by affiliation regime and department of origin in Colombia during the period 2011-2012 | Castañeda, 2016 | Thesis |
| 263 | Qualitative analysis of maternal deaths in Buenaventura 2010 - 2012 with a focus on social determinants and equity | Soto, 2016 | Thesis |
| 264 | Characterization of patients diagnosed with breast cancer, at the hospital de la samaritana, bogota 2010 - 2018. | Peñafiel, 2020 | Thesis |
| 265 | Barriers to access to the Expanded Immunization Program, of the child population attending the neighborhood houses of Tibabitá and Horizontes in the town of Usaquén, Bogotá DC, year 2008 | Becerra, 2011 | Thesis |
| 266 | Equity in health: case study in the capital district of bogota | Martinez, 2006 | Thesis |
| 267 | Factors associated with the use of the emergency service at Hospital San Vicente de Arauca between the period September 2013 and August 2014 | Medina, 2018 | Article |
| 268 | Scale of vulnerability to death due to acute respiratory infection, in children under one year of age, in Bogotá according to social determinants of health | Nuñez, 2017 | Thesis |
