# Supplementary material for: Characterization of the population affiliated to the subsidized health insurance scheme in Colombia: a systematic review and meta-analysis
Source: Int J Equity Health. 2023 Feb 7;22:28. doi: 10.1186/s12939-022-01818-x (PMC9903445; doi:10.1186/s12939-022-01818-x)
Supplement: Supplementary file 2 — Additional file 2: Table 1. Noncommunicable diseases. Table 2. Communicable diseases. Table 3. Obstetric and maternal conditions. Table 4. Frequent conditions in early childhood. Table 5. Other diseases. Illustration 1. Forest plot of cumulative incidence of noncommunicable diseases in the SS, 2016-2021. Illustration 2. Forest Plot of cumulative incidence of communicable disease. Illustration 3. Forest plot of cumulative incidence of obstetric and maternal conditions in the SS, 2018-2021. Illustration 4. Forest plot of cumulative incidence of common conditions in early childhood in the SS, 2009-2021. Illustration 5. Forest plot of cumulative incidence of other diseases in SS, 2015-2021. Illustration 6. Forest plot of cumulative incidence of non-communicable diseases in the CS, 2016-2021. Illustration 7. Forest plot of cumulative incidence of CS communicable diseases, 2012-2021. Illustration 8. Forest plot of cumulative incidence of obstetric and maternal conditions in CS, 2018-2021. Illustration 9. Forest plot of cumulative incidence of common conditions in early childhood in the CS, 2009-2021. Illustration 10. Forest plot of cumulative incidence of others in CS, 2015-2021. [file 12939_2022_1818_MOESM2_ESM.docx]

### Additional file 2

### Cumulative incidences by disease group

Table 1. Noncommunicable diseases

| **Healthy conditions** | **Author, year** | **SS incidence** | **95% CI** | | **CS incidence** | **95% CI** | |
| --- | --- | --- | --- | --- | --- | --- | --- |
| Cancer | High Cost Account, 2020 | 0.003194 | 0.003172 | 0.003217 | 0.008464 | 0.008425 | 0.008502 |
| Rheumatoid arthritis | High Cost Account, 2020 | 0.000990 | 0.000978 | 0.001003 | 0.003274 | 0.003251 | 0.003299 |
| Cancer | INS, 2016 | 0.000090 | 0.000086 | 0.000094 | 0.000128 | 0.000123 | 0.000133 |
| Transplants | INS, 2020 | 0.000009 | 0.000008 | 0.000011 | 0.000025 | 0.000023 | 0.000027 |
| orphan diseases | INS, 2021 | 0.000464 | 0.000455 | 0.000473 | 0.001506 | 0.001491 | 0.001522 |
| Cancer in children under 18 years of age | INS, 2019 | 0.000037 | 0.000034 | 0.000039 | 0.000039 | 0.000036 | 0.000041 |
| Transplants | INS, 2018 | 0.000012 | 0.000011 | 0.000013 | 0.000038 | 0.000036 | 0.000041 |
| Malnutrition | INS, 2019 | 0.000513 | 0.000503 | 0.000522 | 0.000166 | 0.000161 | 0.000172 |
| Kidney transplants | INS, 2016 | 0.000005 | 0.000004 | 0.000006 | 0.000022 | 0.000020 | 0.000024 |
| Malnutrition | INS, 2018 | 0.000464 | 0.000455 | 0.000473 | 0.000155 | 0.000150 | 0.000161 |
| Transplants | INS, 2021 | 0.000003 | 0.000002 | 0.000004 | 0.000010 | 0.000009 | 0.000012 |
| heart transplants | INS, 2019 | 0.000001 | 0.000000 | 0.000001 | 0.000002 | 0.000002 | 0.000003 |
| Liver transplant | INS, 2019 | 0.000002 | 0.000001 | 0.000002 | 0.000006 | 0.000005 | 0.000008 |
| Kidney transplants | INS, 2019 | 0.000008 | 0.000007 | 0.000009 | 0.000024 | 0.000022 | 0.000026 |
| Kidney-pancreas transplant | INS, 2019 | 0.000000 | 0.000000 | 0.000000 | 0.000000 | 0.000000 | 0.000001 |
| Transplants | INS, 2020 | 0.000004 | 0.000003 | 0.000005 | 0.000006 | 0.000005 | 0.000007 |
| Exposure to fluoride | INS, 2018 | 0.000387 | 0.000379 | 0.000395 | 0.000153 | 0.000148 | 0.000159 |
| Transplants | INS, 2018 | 0.000012 | 0.000011 | 0.000014 | 0.000039 | 0.000037 | 0.000042 |
| heart transplants | INS, 2017 | 0.000000 | 0.000000 | 0.000000 | 0.000003 | 0.000002 | 0.000003 |
| lung transplant | INS, 2017 | 0.000000 | 0.000000 | 0.000000 | 0.000001 | 0.000001 | 0.000001 |
| Liver transplant | INS, 2017 | 0.000002 | 0.000001 | 0.000002 | 0.000009 | 0.000008 | 0.000010 |
| Living donor liver transplant | INS, 2017 | 0.000001 | 0.000001 | 0.000001 | 0.000001 | 0.000000 | 0.000001 |
| kidney transplants | INS, 2017 | 0.000009 | 0.000008 | 0.000011 | 0.000027 | 0.000025 | 0.000029 |
| Living donor kidney transplants | INS, 2017 | 0.000001 | 0.000000 | 0.000001 | 0.000005 | 0.000005 | 0.000007 |
| orphan diseases | INS, 2020 | 0.000074 | 0.000071 | 0.000077 | 0.000271 | 0.000264 | 0.000278 |

INS: National Institute of Health, CS: contributory scheme, SS: subsidized scheme, CI: confidence interval

Table 2. Communicable diseases

| **Healthy conditions** | **Author, year** | **SS incidence** | **95% CI** | | **CS incidence** | **95% CI** | |
| --- | --- | --- | --- | --- | --- | --- | --- |
| HIV AIDS | INS, 2020 | 0.000213 | 0.000207 | 0.000218 | 0.000285 | 0.000278 | 0.000292 |
| HIV AIDS | INS, 2018 | 0.000262 | 0.000256 | 0.000269 | 0.000337 | 0.000329 | 0.000345 |
| HIV AIDS | INS, 2016 | 0.000216 | 0.000210 | 0.000222 | 0.000272 | 0.00265 | 0.000279 |
| HIV AIDS | INS, 2015 | 0.000188 | 0.0000183 | 0.000194 | 0.000241 | 0.000235 | 0.000248 |
| HIV AIDS | INS, 2012 | 0.000149 | 0.000144 | 0.000155 | 0.000167 | 0.000162 | 0.000173 |
| Chickenpox | INS, 2019 | 0.001224 | 0.001210 | 0.001239 | 0.001571 | 0.001555 | 0.001588 |
| Chickenpox | INS, 2018 | 0.001382 | 0.001367 | 0.001397 | 0.001859 | 0.001841 | 0.001877 |
| Tuberculosis | INS, 2019 | 0.000008 | 0.000007 | 0.000009 | 0.000005 | 0.000004 | 0.000006 |
| Tuberculosis | INS, 2018 | 0.000010 | 0.000009 | 0.000011 | 0.000006 | 0.000005 | 0.000007 |
| Tuberculosis | INS, 2019 | 0.000356 | 0.000348 | 0.000364 | 0.000225 | 0.000219 | 0.000231 |
| Whooping cough | INS, 2019 | 0.000000 | 0.000000 | 0.000001 | 0.000086 | 0.000083 | 0.000090 |
| Whooping cough | INS, 2018 | 0.000000 | 0.000000 | 0.000001 | 0.000104 | 0.000100 | 0.000109 |
| Accidental tetanus | INS, 2019 | 0.000000 | 0.000000 | 0.000000 | 0.000000 | 0.000000 | 0.000001 |
| Accidental tetanus | INS, 2018 | 0.000089 | 0.000085 | 0.000093 | 0.000000 | 0.000000 | 0.000000 |
| ESAVI | INS, 2018 | 0.000092 | 0.000088 | 0.000096 | 0.000009 | 0.000008 | 0.000010 |
| congenital rubella | INS, 2020 | 0.000000 | 0.000000 | 0.000000 | 0.000014 | 0.000012 | 0.000016 |
| congenital rubella | INS, 2019 | 0.000000 | 0.000000 | 0.000000 | 0.000010 | 0.000009 | 0.000012 |
| Aggressions by animals transmitting rabies | INS, 2019 | 0.000001 | 0.000001 | 0.000002 | 0.003109 | 0.003086 | 0.003132 |
| Ophidic accident | INS, 2019 | 0.000000 | 0.000000 | 0.000000 | 0.000037 | 0.000035 | 0.000040 |
| Ophidic accident | INS, 2018 | 0.000000 | 0.000000 | 0.000001 | 0.000036 | 0.000034 | 0.000039 |
| Chikungunya | INS, 2014 | 0.000002 | 0.000001 | 0.000002 | 0.000018 | 0.000016 | 0.000020 |
| congenital rubella | INS, 2018 | 0.000006 | 0.000005 | 0.000007 | 0.000008 | 0.000007 | 0.000009 |
| Congenital syphilis | INS, 2020 | 0.000006 | 0.000005 | 0.000007 | 0.000007 | 0.000006 | 0.000008 |
| Congenital syphilis | INS, 2018 | 0.000004 | 0.000004 | 0.000005 | 0.000007 | 0.000006 | 0.000008 |
| measles and rubella | INS, 2019 | 0.002782 | 0.002761 | 0.002804 | 0.000110 | 0.000106 | 0.000114 |
| measles and rubella | INS, 2018 | 0.000186 | 0.000180 | 0.000191 | 0.000026 | 0.000024 | 0.000028 |
| measles and rubella | INS, 2018 | 0.000185 | 0.000179 | 0.000190 | 0.000189 | 0.000184 | 0.000195 |
| measles and rubella | INS, 2020 | 0.000135 | 0.000130 | 0.000140 | 0.000016 | 0.000014 | 0.000017 |
| Parotitis | INS, 2019 | 0.000005 | 0.000005 | 0.000006 | 0.000447 | 0.000438 | 0.000456 |
| Parotitis | INS, 2018 | 0.000007 | 0.000006 | 0.000008 | 0.000606 | 0.000596 | 0.000616 |
| Acute flaccid paralysis | INS, 2020 | 0.000029 | 0.000027 | 0.000031 | 0.000002 | 0.000001 | 0.000002 |
| Acute flaccid paralysis | INS, 2018 | 0.000046 | 0.000043 | 0.000049 | 0.000003 | 0.000002 | 0.000004 |
| Acute flaccid paralysis | INS, 2019 | 0.000006 | 0.000005 | 0.000007 | 0.000002 | 0.000002 | 0.000003 |
| Bacterial meningitis | INS, 2020 | 0.000076 | 0.000072 | 0.000079 | 0.000009 | 0.000008 | 0.000011 |
| Meningococcal disease | INS, 2020 | 0.000007 | 0.000006 | 0.000009 | 0.000001 | 0.000001 | 0.000002 |
| Meningococcal disease | INS, 2018 | 0.000162 | 0.000157 | 0.000167 | 0.000002 | 0.000002 | 0.000003 |
| Bacterial meningitis | INS, 2018 | 0.000220 | 0.000214 | 0.000226 | 0.000010 | 0.000008 | 0.000011 |
| Diphtheria | INS, 2018 | 0.000003 | 0.000002 | 0.000003 | 0.000001 | 0.000001 | 0.000002 |
| Dengue | INS, 2018 | 0.000004 | 0.000003 | 0.000005 | 0.000398 | 0.000398 | 0.000406 |
| Leprosy | INS, 2019 | 0.000004 | 0.000003 | 0.000005 | 0.000005 | 0.000004 | 0.000006 |
| Leishmaniasis | INS, 2019 | 0.000011 | 0.000010 | 0.000013 | 0.000039 | 0.000036 | 0.000042 |
| Leprosy | INS, 2018 | 0.000002 | 0.000002 | 0.000003 | 0.000005 | 0.000004 | 0.000006 |
| Letospirosis | INS, 2019 | 0.000002 | 0.000002 | 0.000003 | 0.000002 | 0.000001 | 0.000002 |
| Letospirosis | INS, 2015 | 0.000012 | 0.000010 | 0.000013 | 0.000035 | 0.000032 | 0.000037 |
| Hepatitis A | INS, 2018 | 0.000001 | 0.000001 | 0.000001 | 0.000050 | 0.000047 | 0.000053 |
| Severe respiratory insufficiency | INS, 2012 | 0.000474 | 0.000465 | 0.000483 | 0.000035 | 0.000032 | 0.000038 |
| Yellow fever | INS, 2018 | 0.000011 | 0.000009 | 0.000012 | 0.000000 | 0.000000 | 0.000001 |
| Chikungunya | INS, 2018 | 0.000176 | 0.0000171 | 0.000182 | 0.000013 | 0.000011 | 0.000014 |
| Typhoid and paratyphoid fever | INS, 2021 | 0.000011 | 0.000010 | 0.000013 | 0.000000 | 0.000000 | 0.000001 |
| Letospirosis | INS, 2018 | 0.000002 | 0.000001 | 0.000002 | 0.001085 | 0.001071 | 0.001099 |
| Hepatitis B | INS, 2018 | 0.000044 | 0.000042 | 0.000047 | 0.000013 | 0.000012 | 0.000015 |
| Diphtheria | INS, 2020 | 0.000000 | 0.000000 | 0.000001 | 0.000044 | 0.000041 | 0.000047 |
| Dengue | INS, 2019 | 0.000036 | 0.000034 | 0.000039 | 0.000023 | 0.000021 | 0.000025 |
| Chikungunya | INS, 2019 | 0.000062 | 0.000059 | 0.000066 | 0.000061 | 0.000057 | 0.000064 |
| Infections associated with surgical medical procedures | INS, 2018 | 0.000001 | 0.000000 | 0.000001 | 0.000455 | 0.000446 | 0.000464 |
| Zika | INS, 2018 | 0.000007 | 0.000006 | 0.000008 | 0.000001 | 0.000000 | 0.000001 |
| Infections associated with surgical medical procedures | INS, 2019 | 0.000000 | 0.000000 | 0.000001 | 0.000000 | 0.000000 | 0.000001 |
| Acute respiratory infections- unusual SARI | INS, 2020 | 0.000044 | 0.000042 | 0.000047 | 0.000021 | 0.000019 | 0.000023 |
| Diphtheria | INS, 2019 | 0.000041 | 0.000039 | 0.000044 | 0.000000 | 0.000000 | 0.000001 |
| Equine encephalitis | INS, 2020 | 0.000000 | 0.000000 | 0.000000 | 0.000001 | 0.000001 | 0.000002 |
| Acute respiratory infections- unusual SARI | INS, 2019 | 0.000001 | 0.000001 | 0.000002 | 0.000000 | 0.000000 | 0.000000 |
| Acute chagas | INS, 2018 | 0.000046 | 0.000043 | 0.000049 | 0., 000000 | 0.000000 | 0.000000 |
| Chronic chagas | INS, 2018 | 0.000013 | 0.000011 | 0.000014 | 0.000003 | 0.000003 | 0.000004 |
| Malaria | INS, 2015 | 0.000058 | 0.000055 | 0.000061 | 0.000008 | 0.000006 | 0.000009 |
| Malaria | INS, 2014 | 0.000377 | 0.000370 | 0.000385 | 0.000005 | 0.000005 | 0.000006 |
| Zika | INS, 2020 | 0.000001 | 0.000001 | 0.000001 | 0.000003 | 0.000003 | 0.000004 |
| ESAVI | INS, 2020 | 0.000000 | 0.000000 | 0.000001 | 0.000008 | 0.000006 | 0.000009 |
| ESAVI | INS, 2021 | 0.000013 | 0.000012 | 0.000015 | 0., 000005 | 0.000005 | 0.000006 |
| Chikungunya | INS, 2021 | 0.000000 | 0.000000 | 0.000001 | 0., 000004 | 0.000005 | 0.000006 |
| Malaria | INS, 2017 | 0.000013 | 0.000011 | 0.000014 | 0.000009 | 0.000007 | 0.000010 |
| Zika | Ruiz, 2017 | 0.000000 | 0.000000 | 0.000001 | 0.120000 | 0.107414 | 0.133840 |
| Dengue, chickenpox and malaria | Hilarion, 2019 | 0.470235 | 0.469003 | 0.471467 | 0.421670 | 0.420451 | 0.422889 |

INS: National Institute of Health, CS: contributory regimen, SS: subsidized regimen, CI: confidence interval, HIV: human immunodeficiency virus, AIDS: adult immunodeficiency syndrome, ESAVI: adverse event supposedly attributable to vaccination and immunization

Table 3. Obstetric and maternal conditions

| **Healthy conditions** | **Author, year** | **SS incidence** | **95% CI** | | **CS incidence** | **95% CI** | |
| --- | --- | --- | --- | --- | --- | --- | --- |
| Gestational syphilis | INS, 2019 | 0.000189 | 0.000183 | 0.000195 | 0.000060 | 0.000057 | 0.000063 |
| Gestational syphilis | INS, 2020 | 0.000203 | 0.000197 | 0.000209 | 0.000062 | 0.000058 | 0.000065 |
| Gestational syphilis | INS, 2018 | 0.000177 | 0.000171 | 0.000182 | 0.000058 | 0.000055 | 0.000062 |
| Maternal mortality | INS, 2019 | 0.000009 | 0.000008 | 0.000011 | 0.000004 | 0.000004 | 0.000005 |
| Extreme maternal morbidity | INS, 2018 | 0.000537 | 0.000528 | 0.000547 | 0.000443 | 0.000434 | 0.000452 |
| Maternal mortality | INS, 2021 | 0.000008 | 0.000007 | 0.000009 | 0.000005 | 0.000004 | 0.000006 |
| Maternal mortality | INS, 2020 | 0.000011 | 0.000010 | 0.000012 | 0.000004 | 0.000003 | 0.000005 |
| Extreme maternal morbidity | INS, 2019 | 0.000505 | 0.000496 | 0.000514 | 0.000429 | 0.000420 | 0.000437 |
| Puerperal endometritis | INS, 2018 | 0.000020 | 0.000018 | 0.000022 | 0.000021 | 0.000019 | 0.000023 |
| Maternal mortality | INS, 2020 | 0.000005 | 0.000004 | 0.000006 | 0.000002 | 0.000001 | 0.000002 |

INS: National Institute of Health, CS: contributory scheme, SS: subsidized scheme, CI: confidence interval

Table 4. Frequent conditions in early childhood.

| **Healthy conditions** | **Author, year** | **SS incidence** | **95% CI** | | **CS incidence** | **95% CI** | |
| --- | --- | --- | --- | --- | --- | --- | --- |
| Low birth weight | INS, 2019 | 0.000407 | 0.000399 | 0.000415 | 0.000361 | 0.000353 | 0.000369 |
| Low birth weight | INS, 2018 | 0.000446 | 0.000437 | 0.000454 | 0.000376 | 0.000368 | 0.000385 |
| Low birth weight | INS, 2021 | 0.000228 | 0.000222 | 0.000235 | 0.000181 | 0.000176 | 0.000187 |
| Perinatal and late neonatal mortality | INS, 2018 | 0.000266 | 0.000259 | 0.000273 | 0.000143 | 0.000138 | 0.000148 |
| Perinatal and late neonatal mortality | INS, 2019 | 0.000236 | 0.000230 | 0.000242 | 0.000124 | 0.000119 | 0.000128 |
| Congenital malformations | INS, 2010 | 0.373671 | 0.365644 | 0.378726 | 0.313405 | 0.308591 | 0.318258 |
| Congenital defects | INS, 2018 | 0.000149 | 0.000145 | 0.000155 | 0.000132 | 0.000128 | 0.000137 |
| Congenital defects | INS, 2020 | 0.000063 | 0.000060 | 0.000066 | 0.000063 | 0.000059 | 0.000066 |
| Low birth weight | INS, 2020 | 0.000212 | 0.000206 | 0.000218 | 0.000219 | 0.000213 | 0.000226 |
| Low birth weight | daza, 2009 | 0.561064 | 0.0527037 | 0.594527 | 0.064087 | 0.049328 | 0.082877 |
| Neonatal death | Houweling, 2016 | 0.472189 | 0.471571 | 0.472807 | 0.395104 | 0.394499 | 0.395710 |

INS: National Institute of Health, CS: contributory scheme, SS: subsidized scheme, CI: confidence interval

Table 5. Other diseases

| **Healthy conditions** | **Author, year** | **SS incidence** | **95% CI** | | **CS incidence** | **95% CI** | |
| --- | --- | --- | --- | --- | --- | --- | --- |
| Gender and domestic violence | INS, 2019 | 0.002762 | 0.002740 | 0.002783 | 0.001889 | 0.001871 | 0.001907 |
| External cause by aesthetic procedure | INS, 2020 | 0.000001 | 0.000001 | 0.000002 | 0.000004 | 0.000004 | 0.000003 |
| External cause for consumer accidents | INS, 2020 | 0.000006 | 0.000005 | 0.000007 | 0.000003 | 0.000002 | 0.000004 |
| External cause for consumer accidents | INS, 2021 | 0.000001 | 0.000001 | 0.000002 | 0.000001 | 0.000000 | 0.000001 |
| External cause by aesthetic procedure | INS, 2021 | 0.000000 | 0.000000 | 0.000001 | 0.000001 | 0.000001 | 0.000002 |
| Poisonings | INS, 2019 | 0.000482 | 0.000473 | 0.000491 | 0.000457 | 0.000448 | 0.000466 |
| Poisonings | INS, 2018 | 0.000489 | 0.000480 | 0.000499 | 0.000489 | 0.000480 | 0.000499 |
| External cause for consumer accidents | INS, 2018 | 0.000042 | 0.000040 | 0.000045 | 0.000015 | 0.000014 | 0.000017 |
| External cause by aesthetic procedure | INS, 2018 | 0.000004 | 0.000003 | 0.000005 | 0.000014 | 0.000012 | 0.000015 |
| Sexual abuse | Moran, 2017 | 0.535280 | 0.535280 | 0.627998 | 0.354988 | 0.311276 | 0.401263 |
| Maxillofacial trauma | Pineda, 2015 | 0.503516 | 0.503516 | 0.612529 | 0.888889 | 0.062215 | 0.125469 |

INS: National Institute of Health, CS: contributory scheme, SS: subsidized scheme, CI: confidence interval

**Forest plot of cumulative incidents for the subsidized and contributory regime**

Subsidized scheme

Ilustration 1. Forest plot of cumulative incidence of noncommunicable diseases in the SS, 2016-2021

Illustration 2 . Forest plot of cumulative incidence of transmissible diseases in the SS, 2012-2021

Illustration 3 . Forest plot of cumulative incidence of obstetric and maternal conditions in the SS, 2018-2021

Illustration 4 . Forest plot of cumulative incidence of common conditions in early childhood in the SS, 2009-2021
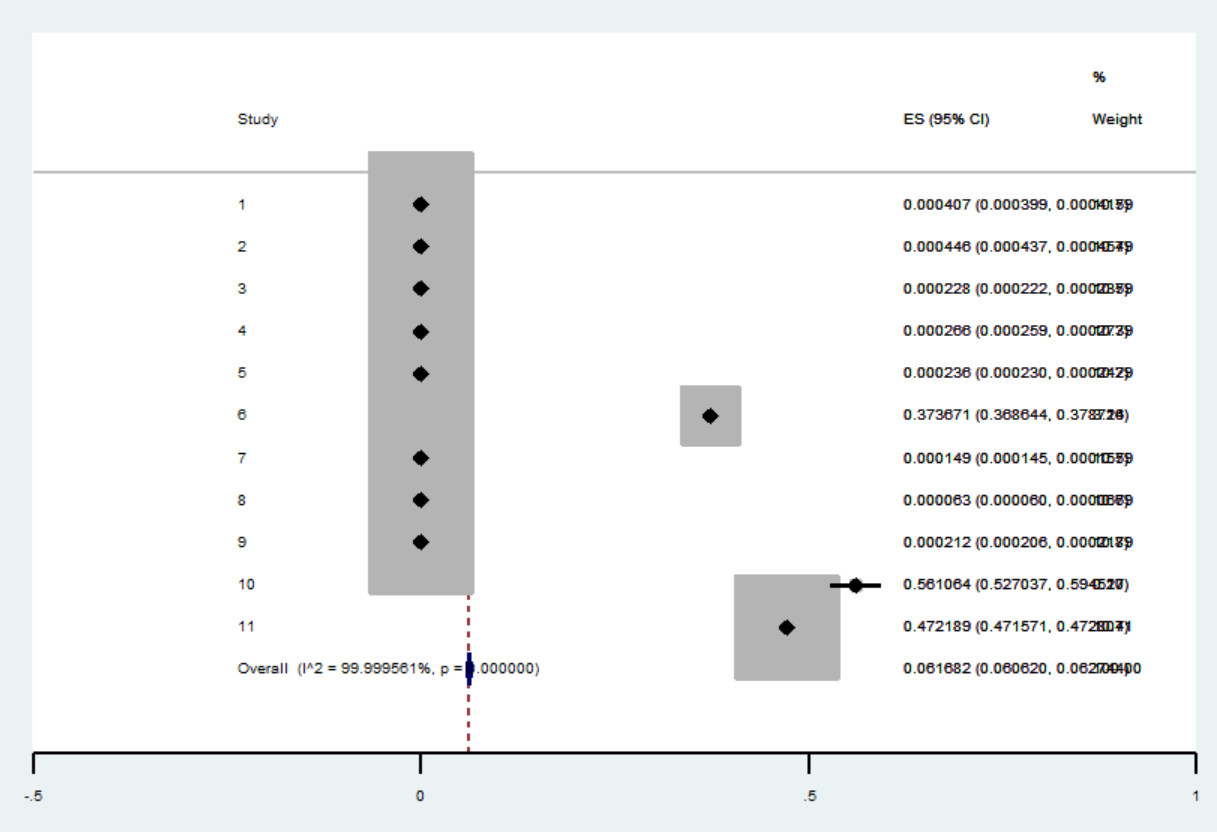


Illustration 5. Forest plot of cumulative incidence of other diseases in SS, 2015-2021

**Contributory scheme**

Illustration 6 . Forest plot of cumulative incidence of non-communicable diseases in the CS, 2016-2021

Illustration 7 . Forest plot of cumulative incidence of CS communicable diseases, 2012-2021

Illustration 8 . Forest plot of cumulative incidence of obstetric and maternal conditions in CS, 2018-2021

Illustration 9 . Forest plot of cumulative incidence of common conditions in early childhood in the CS, 2009-2021

Illustration 10 . Forest plot of cumulative incidence of others in CS, 2015-2021
