# Supplementary material for: Characterization of the population affiliated to the subsidized health insurance scheme in Colombia: a systematic review and meta-analysis
Source: Int J Equity Health. 2023 Feb 7;22:28. doi: 10.1186/s12939-022-01818-x (PMC9903445; doi:10.1186/s12939-022-01818-x)
Supplement: Supplementary file 3 — Additional file 3: Table 1. Distribution of cases by regimen in morbid cohorts of non-communicable diseases between 2004 and 2020. Table 2. Description of studies with information on communicable diseases from the SS, 2018-2021. Table 3. Distribution of cases between regimens. Health services. 2009-2021. Table 4. Use of health services. Table 5. Distribution of cases by regimen in morbid cohorts in health services. 2009-2019. [file 12939_2022_1818_MOESM3_ESM.docx]

Additional file 3.

Distribution of cases wether subsidiary scheme or contributory scheme

Table*1*. Distribution of cases by regimen in morbid cohorts of non-communicable diseases between 2004-2020

| **Author, year** | **Health condition** | **Data source** | **SS cases** | **CS cases** | **Total** | **SS/CS ratio** |
| --- | --- | --- | --- | --- | --- | --- |
| Echeverria, 2011 (253) | Dental conditions (cavities, use of amalgams, prophylaxis) | Fontibon Hospital | 22 | 188 | 265 | 0.117 |
| Egurrola-Pedraza, 2019 (29) | Breast cancer | Institute of Cancerology (IoC) The Americas | 385 | 2,347 | 2,732 | 0.164 |
| Martinez Perez, 2019 (28) | Breast cancer | Las Américas Cancer Institute, Pablo Tobón Uribe Hospital, Antioquia Cancer Center and Vida Clinic | 36 | 206 | 242 | 0.175 |
| Pineros, 2011 (67) | Breast cancer | Institutions that provided oncological services for this cancer in 2006. | 307 | 1,291 | 1,684 | 0.238 |
| Atehortua,2014(230) | Cervical cancer | Colombia Quality of Life Survey 2008 | 5,099 | 20,925 | 33,188 | 0.244 |
| Novoa, 2019 (262) | Asthma | Colombian Neumological Foundation | eleven | 44 | 56 | 0.250 |
| Posada, 2016 (256) | Oral cancer | Specialized cancer centers | 153 | 404 | 630 | 0.379 |
| Borda, 2018 (72) | Prostate cancer | Survey Health, Well-being and Aging in Latin America and the Caribbean | 201 | 507 | 732 | 0.397 |
| Osorio, 2015 (32) | Dental disease / cavities | Community | 131 | 307 | 483 | 0.427 |
| Vergara, 2020(255) | Colorectal cancer | San Diego Clinical Cancer Research Center and ConvaTec Medical Care | 33 | 69 | 104 | 0.478 |
| Barrier, 2017(232) | odontology | Santander Public Health Observatory (SPHO) | 2,309 | 4,563 | 8,692 | 0.506 |
| Duke, 2019 (249) | Cerebrovascular disease | Comprehensive Social Protection Information System (CSPIS) | 24,309 | 44,594 | 75,905 | 0.545 |
| Devries, 2015 (20) | gastric cancer | Cancer Registry | 344 | 550 | 1,039 | 0.625 |
| Gomez, 2020(25) | Acute lymphoid leukemia | Public Health Surveillance System (SIVIGILA) | 72 | 109 | 190 | 0.661 |
| Charry, 2009 (44) | Breast cancer | Institutions authorized for the treatment of breast cancer | 298 | 411 | 806 | 0.725 |
| Charlie, 2008(46) | Breast cancer | Public and private institutions | 302 | 416 | 806 | 0.726 |
| Castillo, 2013 (70) | Cervical cancer | Community | 381 | 516 | 915 | 0.738 |
| Jaramillo, 2004(64) | Cancer | DANE | 59,369 | 76,605 | 19.1513 | 0.775 |
| Baquero, 2010 (78) | Cancer | Subsidized and contributory insurers and the uninsured poor population | 97 | 122 | 256 | 0.795 |
| Garces-Palacios, 2010(265) | cervical cancer | demographic and health survey | 8,416 | 9,393 | 17,809 | 0.896 |
| Torres, 2020 (226) | Colorectal cancer | National Cancer Institute | 139 | 154 | 300 | 0.903 |
| Barahona, 2020 (264) | Rheumatoid arthritis | Health institutions | 80 | 80 | 240 | 1,000 |
| Heads, 2011 (40) | Chronic disease | Consult a government hospital or clinic,  government health center or post,  hospital or private clinic,  center of attention of an HPE /  private medical office | 30,082 | 29,692 | 75,071 | 1,013 |
| Garcia, 2020 (251) | Acute lymphoid leukemia | DANE and SIVIGILA | 954 | 906 | 2,082 | 1,053 |
| Rodriguez, 2017 (231) | Cardiovascular diseases | CSPIS | 295,218 | 222,708 | 517,926 | 1,326 |
| Pinilla, 2011 (26) | Arterial hypertension | External consultation of different institutions of the SS and CS | 79 | 57 | 136 | 1,386 |
| Pastrán, 2017 (65) | Disability | Community | 142 | 85 | 231 | 1,671 |
| Hernandez, 2019 (56) | Skin lesions | Private clinic and public hospital | 231 | 123 | 409 | 1,878 |
| Arango, 2012 (73) | Mortality due to nutritional deficiencies | DANE | 213 | 107 | 989 | 1,991 |
| Ducuara, 2012 (243) | Malnutrition | Quality of Life Survey for Bogotá 2007 | 2,237 | 924 | 3,498 | 2,421 |
| Soto, 2019 (238) | epilespsies | San Rafael Hospital | 46 | 12 | 58 | 3,833 |
| Perez, 2021 (247) | Chronic diseases | Peter Hospital  Claver Aguirre | 704 | 124 | 847 | 5,677 |
| Penafiel, 2020 (224) | Breast cancer | Samaritana Hospital | 223 | one | 240 | 223,000 |

SS: subsidized scheme, CS: contributory scheme, DANE: National Administrative Department of Statistics, Institute of Cancerology (IoC), Santander Public Health Observatory (SPHO), Comprehensive Social Protection Information System (CSPIS), Health Promoting Entities: HPE

Table*2*. Description of studies with information on communicable diseases from the SS, 2018-2021

| **Author, Year** | **Document type** | **Nature** | **Data source** | **Health condition** | **Total** | **SS cases** | **%** |
| --- | --- | --- | --- | --- | --- | --- | --- |
| INS, 2020 | Event report | Public | INS | Leptospirosis | 1,240 | 24 | 1.94 |
| INS, 2020 | Event report | Public | INS | Ophidic accident | 3,303 | 260 | 7.87 |
| INS, 2021 | Infographic | Public | INS | Ophidic accident | 2,719 | 251 | 9.20 |
| INS, 2020 | Event report | Public | INS | Accidental tetanus | 9 | two | 22.22 |
| INS, 2021 | Infographic | Public | INS | Acute chagas | 56 | 19 | 33.93 |
| INS, 2020 | Event report | Public | INS | Dengue | 68,226 | 30,590 | 44.84 |
| INS, 2020 | Event report | Public | INS | infections associated with surgical medical procedures | 800 | 361 | 45.13 |
| INS, 2021 | Infographic | Public | INS | Letospirosis | 1,246 | 568 | 45.59 |
| INS, 2021 | Infographic | Public | INS | Yellow fever | 12 | 6 | 50.00 |
| INS, 2020 | Event report | Public | INS | Congenital syphilis | 1,087 | 544 | 50.00 |
| INS, 2019 | Event report | Public | INS | Neonatal tetanus | 2 | 1 | 50.00 |
| INS, 2021 | Infographic | Public | INS | Dengue | 21,112 | 10,591 | 50.17 |
| INS, 2021 | Event report | Public | INS | infections associated with surgical medical procedures | 1,765 | 899 | 50.93 |
| INS, 2020 | Event report | Public | INS | infections associated with surgical medical procedures | 1,765 | 899 | 50.93 |
| INS, 2021 | Infographic | Public | INS | Leprosy | 125 | 72 | 57.60 |
| INS, 2021 | Event report | Public | INS | Congenital syphilis | 1,132 | 659 | 58.20 |
| INS, 2021 | Infographic | Public | INS | Whooping cough | 831 | 491 | 59.10 |
| INS, 2021 | Infographic | Public | INS | Zika | 57 | 35 | 61.40 |
| INS, 2021 | Infographic | Public | INS | Mucous leishmaniasis | 30 | 20 | 67.30 |
| INS, 2018 | Event report | Public | INS | Equine encephalitis | 8 | 6 | 75.00 |
| INS, 2020 | Event report | Public | INS | Accidental tetanus | 25 | 19 | 76.00 |
| INS, 2018 | Event report | Public | INS | Malaria | 63,143 | 48,494 | 76.80 |
| INS, 2021 | Infographic | Public | INS | Malaria | 43,590 | 33,899 | 77.80 |
| INS, 2017 | Outbreak study | Public | INS | Leishmaniasis | 8 | 7 | 87.50 |
| INS, 2018 | Event report | Public | INS | trachoma | 9 | 8 | 88.89 |
| INS, 2019 | Event report | Public | INS | trachoma | 5 | 5 | 100.00 |
| INS, 2020 | Infographic | Public | INS | trachoma | 1 | 1 | 100.00 |
| INS, 2021 | Infographic | Public | INS | Neonatal tetanus | 3 | 3 | 100.00 |
| INS, 2020 | Event report | Public | INS | Neonatal tetanus | 2 | 2 | 100.00 |
| INS, 2018 | Event report | Public | INS | Neonatal tetanus | 5 | 5 | 100.00 |

 SS: subsidized scheme, CS: contributory scheme.

Table*3*. Distribution of cases between regimens. Health services. 2009-2021

| **Author, year** | **Data source** | **Type of service** | **People affiliated with the SR in the study** | **Number of people who demanded attention in the SS** | **Frequency of use of SS service (%)** | **People affiliated with the CS in the study** | **Number of people who demanded attention in the CR** | **Frequency of use of CS services (%)** | **OR** | **SS/CS prevalence ratio** | **P-value** |
| --- | --- | --- | --- | --- | --- | --- | --- | --- | --- | --- | --- |
| INS, 2016 | INS | Use of radiation therapy | 2,025 | 153 | 7.6 | 2,782 | 149 | 5.4 | 1.4 | 1.4 | 0.002 |
|  |  | Use of chemotherapy and radiation therapy | 2,025 | 93 | 4.6 | 2,782 | 97 | 3.5 | 1.3 | 1.3 | 0.052 |
| Pineros, 2011 | Institutions with oncology services /Bogota | Diagnosis of breast cancer | 307 | 137 | 44.6 | 1,291 | 567 | 43.9 | 1.0 | 1.0 | 0.823 |
|  |  | Breast cancer treatment | 307 | 135 | 44.0 | 1,291 | 554 | 42.9 | 1.0 | 1.0 | 0.736 |
| Murica, 2014 | Hospital Universitario del  Valle (HUV), and Fundación Valle del Lili (FVL), | Patients with prenatal ultrasound | 246 | 110 | 44.7 | 115 | 82 | 71.3 | 0.3 | 0.6 | 0.000 |
|  |  | Patients without prenatal ultrasound | 246 | 136 | 55.3 | 115 | 33 | 28.7 | 3.1 | 1.9 | 0.000 |
| Castle, 2013 | Community / Cartagena | Compliance with the cytology scheme according to the norm | 381 | 107 | 28.1 | 516 | 206 | 39.9 | 0.6 | 0.7 | 0.000 |
| Echeverria, 2011 | Fontibon Hospital | Completion of dental treatments | 22 | 6 | 27.3 | 188 | 83 | 44.1 | 0.5 | 0.6 | 0.130 |
| Towers, 2020 | National Cancer Institute / Colombia | In-hospital management | 300 | 138 | 46.0 | 154 | 153 | 99.4 | 0.006 | 0.5 | 0.000 |
|  |  | Treatment: observationoneand monitoring | 300 | 16 | 5.3 | 154 | 16 | 10.4 | 0.5 | 0.5 | 0.046 |
|  |  | Neoadjuvant and surgery | 300 | 5 | 1.7 | 154 | 6 | 3.9 | 0.4 | 0.4 | 0.144 |
|  |  | Neoadjuvant and surgery and adjuvant | 300 | 17 | 5.7 | 154 | 14 | 9.1 | 0.6 | 0.6 | 0.171 |
|  |  | Surgery and adjuvant | 300 | 12 | 4.0 | 154 | 29 | 18.8 | 0.2 | 0.2 | 0.000 |
|  |  | Surgery | 300 | twenty | 6.7 | 154 | 31 | 20.1 | 0.3 | 0.3 | 0.000 |
|  |  | Radiotherapy | 300 | 62 | 20.7 | 154 | 46 | 29.9 | 0.6 | 0.7 | 0.029 |
|  |  | Chemotherapy | 300 | 97 | 32.3 | 154 | 100 | 64.9 | 0.3 | 0.5 | 0.000 |
|  |  | Palliative care | 300 | 37 | 12.3 | 154 | 3. 4 | 22.1 | 0.5 | 0.6 | 0.007 |
|  |  | Diagnostic colonoscopy | 300 | 111 | 37.0 | 154 | 118 | 76.6 | 0.2 | 0.5 | 0.000 |
|  |  | Psychological evaluationone | 300 | 30 | 10.0 | 154 | 29 | 18.8 | 0.5 | 0.5 | 0.008 |
|  |  | Evaluation with social work | 300 | 116 | 38.7 | 154 | 116 | 75.3 | 0.2 | 0.5 | 0.000 |
| Egurrola-Pedraza, 2019 | Las Américas Cancer Institute / Medellin | Mammography | 385 | 278 | 72.2 | 2,347 | 1,987 | 84.7 | 0.5 | 0.9 | 0.000 |
|  |  | Radiotherapy | 385 | 280 | 72.7 | 2,347 | 1,752 | 74.6 | 0.9 | 1.0 | 0.423 |
|  |  | Chemotherapy | 385 | 276 | 71.7 | 2,347 | 1,418 | 60.4 | 1.7 | 1.2 | 0.000 |
|  |  | biological therapyone | 385 | 52 | 13.5 | 2,347 | 315 | 13.4 | 1.0 | 1.0 | 0.964 |
|  |  | Mastectomy | 385 | 266 | 69.1 | 2,347 | 1,041 | 44.4 | 2.8 | 1.6 | 0.000 |
| Pinilla, 2011 | External consultation SS and CS institution / Colombia | Prescription of antihypertensives | 79 | 24 | 30.4 | 57 | 25 | 43.9 | 0.6 | 0.7 | 0.106 |
| Barahona, 2020 | CS: private rheumatology outpatient clinic, RS: Network of hospitals in the north of Bogota | Patient with rheumatoid arthitis with high disease activity at the start of follow-up | 80 | 48 | 60.0 | 80 | 73 | 91.3 | 0.1 | 0.7 | 0.000 |
|  |  | Patients with rheumatoid arteitis with high disease activity at the end of follow-up (2 years) | 80 | 52 | 65.0 | 80 | 41 | 51.3 | 1.8 | 1.3 | 0.078 |
| Heads, 2011 | National Health Survey | Consult a government hospital or clinic | 15,935 | 5,157 | 32.4 | 18,306 | 1547 | 8.5 | 5.2 | 3.8 | 0.000 |
|  |  | Government health center or post | 15,935 | 3,692 | 23.2 | 18,306 | 407 | 2.2 | 13.3 | 10.4 | 0.000 |
|  |  | Hospital or private clinic | 15,935 | 250 | 1.6 | 18,306 | 835 | 4.6 | 0.3 | 0.3 | 0.000 |
|  |  | Service center of an EPS/ARS | 15,935 | 4,376 | 27.5 | 18,306 | 13,278 | 72.5 | 0.1 | 0.4 | 0.000 |
|  |  | Private medical office | 15,935 | 1,199 | 7.5 | 18,306 | 13,278 | 970.6 | -0.1 | 0.03 | 0.000 |
| Houweling, 2016 | DANE | Vaginal delivery | 1,183,741 | 776,300 | 65.6 | 990,495 | 505,624 | 51.0 | 1.8 | 1.3 | 0.000 |
|  |  | Caesarean section | 1,183,741 | 407,441 | 34.4 | 990,495 | 484,871 | 49.0 | 0.5 | 0.7 | 0.000 |
| Paniagua, 2021 | SIVIGILA / Antioquia | TB cured | 3,215 | 944 | 29.4 | 3,032 | 921 | 30.4 | 1.0 | 1.0 | 0.382 |
|  |  | TB treatment completed | 3,215 | 933 | 29.0 | 3,032 | 1,490 | 49.1 | 0.4 | 0.6 | 0.000 |
|  |  | Failed treatment | 3,215 | 23 | 0.7 | 3,032 | 17 | 0.6 | 1.3 | 1.3 | 0.444 |
| Soto, 2019 | Hospital San Rafael de Tunja | Diagnosis of refractory epilepsy | 46 | 22 | 47.8 | 12 | two | 16.7 | 4.6 | 2.9 | 0.051 |
| Barrier, 2020 | ENSAB VI study | Attended a dental consultation | 3,456 | 2,066 | 59.8 | 2,223 | 1,379 | 62.0 | 0.9 | 1.0 | 0.090 |
|  |  | Characteristics in the affiliation scheme of the caregiver of the children who attended the consultation | 3,084 | 1,825 | 59.2 | 2,522 | 1,544 | 61.2 | 0.9 | 1.0 | 0.120 |
| Arocha, 2021 | National Survey of Demography and Health (ENDS) of 2010 and the National Survey of Food and Nutritional Situation of Colombia | Abandonment of breastfeeding | 536 | 306 | 57.1 | 360 | 179 | 49.7 | 1.3 | 1.1 | 0.030 |
| Sosa, 2020 | National Demographic and Health Survey 2015 | Taking cytology | 6,240 | 5752 | 92.2 | 1,342 | 1,260 | 93.9 | 0.8 | 1.0 | 0.031 |
| Becerra, 2011 | Tibabitá and Horizons town of Usaquén | Complete vaccination schedule - Horizons tenement house | 49 | 28 | 57.1 | 23 | 18 | 78.3 | 0.4 | 0.7 | 0.082 |
|  |  | Late complete vaccination schedule - Horizons tenement house | 49 | 13 | 26.5 | 23 | 3 | 13.0 | 2.4 | 2.0 | 0.199 |
|  |  | Incomplete vaccination schedule - Horizons tenement house | 49 | two | 4.1 | 23 | 8 | 34.8 | 0.1 | 0.1 | 0.000 |
|  |  | Complete vaccination schedule - tibatita | 47 | 27 | 57.4 | 32 | 24 | 75.0 | 0.5 | 0.8 | 0.109 |
|  |  | Complete late vaccination schedule -tibatita | 47 | 13 | 27.7 | 32 | 4 | 12.5 | 2.7 | 2.2 | 0.107 |
|  |  | Incomplete vaccination schedule - tibatita | 47 | 7 | 14.9 | 32 | 4 | 12.5 | 1.2 | 1.2 | 0.763 |

SS: subsidized scheme, CS: contributory scheme, TB: tuberculosis

Table *4*. Use of health services

| **Author, year** | **Data source** | **Type of service** | **Population affiliated with the SS** | **Number of registered attentions for SS** | **Frequency of use** | **Population affiliated with the CS** | **Number of registered attentions for CS** | **Frequency of use** | **P-value** | **Odds Ratio** | **95% CI** |
| --- | --- | --- | --- | --- | --- | --- | --- | --- | --- | --- | --- |
| Ministry of Health, 2014 | MinHealth | General medicine | 10,858,029 | 9,229,325 | 40.71143 | 11,679,085 | 10,160,804 | 500.31392 | 0.000 | 0.8467 | 0.8447 to 0.8488 |
|  |  | Medication delivery | 10,858,029 | 7,057,719 | 31.13227 | 11,679,085 | 6,773,869 | 333.54262 | 0.000 | 1.3448 | 1.3425 to 1.3471 |
|  |  | Laboratory exams | 10,858,029 | 4,343,212 | 19.15832 | 11,679,085 | 4,905,216 | 241.53086 | 0.000 | 0.9206 | 0.9191 to 0.9222 |
|  |  | Specialized medicine | 10,858,029 | 3,474,569 | 15,32665 | 11,679,085 | 4,321,261 | 212.77719 | 0.000 | 0.8013 | 0.7999 to 0.8027 |
|  |  | Emergencies | 10,858,029 | 2,823,088 | 12.45291 | 11,679,085 | 3,270,144 | 161.02057 | 0.000 | 0.9035 | 0.9018 to 0.9052 |
|  |  | Dentistry | 10,858,029 | 2,605,927 | 11.49499 | 11,679,085 | 3,153,353 | 155.26984 | 0.000 | 0.8538 | 0.8522 to 0.8554 |
|  |  | X-ray and images | 10,858,029 | 1,628,704 | 7.18437 | 11,679,085 | 2,102,235 | 103.51323 | 0.000 | 0.8039 | 0.8021 to 0.8057 |
|  |  | Promotion and prevention | 10,858,029 | 977,223 | 4.31062 | 11,679,085 | 1,051,118 | 51.75661 | 0.000 | 1,0000 | 0.9971 to 1.0029 |
|  |  | Priority appointments | 10,858,029 | 542,901 | 2.39479 | 11,679,085 | 1,167,909 | 57.50735 | 0.000 | 0.4737 | 0.4721 to 0.4753 |
|  |  | Hospitalization | 10,858,029 | 868,642 | 3.83166 | 11,679,085 | 583,954 | 28.75367 | 0.000 | 1.6522 | 1.6465 to 1.6578 |
|  |  | Surgery | 10,858,029 | 651,482 | 2.87375 | 11,679,085 | 700,745 | 34.50441 | 0.000 | 1,0000 | 0.9965 to 1.0035 |
|  |  | Therapies or treatments | 10,858,029 | 325,741 | 1.43687 | 11,679,085 | 700,745 | 34.50441 | 0.000 | 0.4845 | 0.4825 to 0.4866 |
|  |  | Request for medical appointments: personally | 10,858,029 | 8,252,102 | 36.40080 | 11,679,085 | 5,255,588 | 258.78306 | 0.000 | 3.8704 | 3.8634 to 3.8774 |
|  |  | Request for medical appointments: telephone | 10,858,029 | 3,365,989 | 14,84770 | 11,679,085 | 7,357,824 | 362,29629 | 0.000 | 0.2639 | 0.2634 to 0.2643 |
|  |  | Request for medical appointments: internet | 10,858,029 | 434,321 | 1.91583 | 11,679,085 | 1,051,118 | 51.75661 | 0.000 | 0.4213 | 0.4198 to 0.4228 |
|  |  | Form to request procedures in your health company: personally | 10,858,029 | 7,817,781 | 34.48497 | 11,679,085 | 6,423,497 | 316,29041 | 0.000 | 2,1039 | 2,1002 to 2,1076 |
|  |  | Form to request procedures in your health company: telephone | 10,858,029 | 2,063,026 | 9,10020 | 11,679,085 | 4,671,634 | 230.02939 | 0.000 | 0.3519 | 0.3512 to 0.3525 |
|  |  | Form to request procedures in your health company: internet | 10,858,029 | 434,321 | 1.91583 | 11,679,085 | 1,167,909 | 57.50735 | 0.000 | 0.3750 | 0.3737 to 0.3763 |
|  |  | Request form for procedures in your health company: written | 10,858,029 | 108,580 | 0.47896 | 11,679,085 | 233,582 | 11.50147 | 0.000 | 0.4949 | 0.4914 to 0.4985 |
|  |  | They have not requested any procedure from their health company | 10,858,029 | 1,737,285 | 7.66333 | 11,679,085 | 1,635,072 | 80.51029 | 0.000 | 1.1701 | 1.1674 to 1.1728 |

 SS: subsidized scheme, CS: contributory scheme.

Table*5*. Distribution of cases by regimen in morbid cohorts in health services. 2009-2019

| **Author, year** | **Type of service** | **Data source** | **Number of people who requested attention in the SS** | **Number of people who requested attention in the CS** | **Total** | **SS / CS ratio** |
| --- | --- | --- | --- | --- | --- | --- |
| Anaya, 2017 | Vaginal delivery | Public hospital / Pereira | 332 | 61 | 393 | 5.4 |
|  | Caesarean section |  | 363 | 82 | 445 | 4.4 |
| Charlie, 2009 | Use of resection-type interventions | Institutions authorized in the management of breast cancer / Bogota | 145 | 192 | 375 | 0.8 |
|  | Use of radiotherapy-type interventions |  | 120 | 166 | 319 | 0.7 |
|  | Use of chemotherapy-type interventions |  | 176 | 189 | 411 | 0.9 |
|  | Use of hormone-type interventions |  | 62 | 53 | 125 | 1.2 |
| martin perez, 2019 | Patients with delays in care | Las Américas Cancer Institute (IDC), Pablo Tobón Uribe Hospital (HPTU), Antioquia Cancer Center (COA) and Vida Clinic / Medellin | 33 | 98 | 242 | 0.3 |
|  | Patients without delay in care |  | 3 | 108 | 242 | 0.03 |

SS: subsidized scheme, CS: contributory scheme.
