# Supplementary material for: Characterization of the population affiliated to the subsidized health insurance scheme in Colombia: a systematic review and meta-analysis
Source: Int J Equity Health. 2023 Feb 7;22:28. doi: 10.1186/s12939-022-01818-x (PMC9903445; doi:10.1186/s12939-022-01818-x)
Supplement: Supplementary file 4 — Additional file 4. [file 12939_2022_1818_MOESM4_ESM.docx]

**Additional file 4**

**Risk of bias assessment**

| **Cases series** |  |  |  |  |
| --- | --- | --- | --- | --- |
| Reviewer: Laura Mora Moreo |  |  |  |  |
| Date: 22/12/2021 | **Author/ year: Agudelo/ 2016** | | | |
| **Question** | **Yes** | **No** | **Unclear** | **Not applicable** |
| Were there clear criteria for inclusion in the case series? | x |  |  |  |
| Was the condition measured in a standard, reliable way for all participants included in the case series? | x |  |  |  |
| Were valid methods used for identification of the condition for all participants included in the case series? | x |  |  |  |
| Did the case series have consecutive inclusion of participants? | x |  |  |  |
| Did the case series have complete inclusion of participants? | x |  |  |  |
| Was there clear reporting of the demographics of the participants in the study? | x |  |  |  |
| Was there clear reporting of clinical information of the participants? | x |  |  |  |
| Were the outcomes or follow up results of cases clearly reported? | x |  |  |  |
| Was there clear reporting of the presenting site(s)/clinic(s) demographic information? | x |  |  |  |
| Was statistical analysis appropriate? | x |  |  |  |
| **Overall apprraisal** | **Include** | | | |

Cross sectional studies

| **Reviewer: Laura Mora Moreo** |  |  |  |  |  |  |  |  |  |  |  |  |  |  |  |  |  |  |  |  |  |  |  |  |  |  |  |  |  |  |  |  |
| --- | --- | --- | --- | --- | --- | --- | --- | --- | --- | --- | --- | --- | --- | --- | --- | --- | --- | --- | --- | --- | --- | --- | --- | --- | --- | --- | --- | --- | --- | --- | --- | --- |
| **Date: 22/12/2021** | **Author/ year: Ettenberg 2014** | | | | **Anaya 2017** | | | | **Arrivillaga 2009** | | | | **Ayala 2015** | | | | **Alavarez Sierra 2014** | | | | **Benjumea 2015** | | | | **Borrero 2012** | | | | **Cano 2016** | | | |
| **Question** | **Yes** | **No** | **Unclear** | **Not applicable** | **Yes** | **No** | **Unclear** | **Not applicable** | **Yes** | **No** | **Unclear** | **Not applicable** | **Yes** | **No** | **Unclear** | **Not applicable** | **Yes** | **No** | **Unclear** | **Not applicable** | **Yes** | **No** | **Unclear** | **Not applicable** | **Yes** | **No** | **Unclear** | **Not applicable** | **Yes** | **No** | **Unclear** | **Not applicable** |
| Were the criteria for inclusion in the sample clearly defined? | x |  |  |  | x |  |  |  | x |  |  |  | x |  |  |  | x |  |  |  | x |  |  |  | x |  |  |  | x |  |  |  |
| Were the study subjects and the setting described in detail? | x |  |  |  | x |  |  |  | x |  |  |  | x |  |  |  | x |  |  |  | x |  |  |  | x |  |  |  | x |  |  |  |
| Was the exposure measured in a valid and reliable way? | x |  |  |  | x |  |  |  | x |  |  |  | x |  |  |  | x |  |  |  | x |  |  |  | x |  |  |  | x |  |  |  |
| Were objective, standard criteria used for measurement of the condition? | X |  |  |  | x |  |  |  | x |  |  |  | x |  |  |  | x |  |  |  | x |  |  |  |  |  | x |  | x |  |  |  |
| Were confounding factors identified? | x |  |  |  | x |  |  |  | x |  |  |  |  | x |  |  |  | x |  |  |  | x |  |  |  | x |  |  | x |  |  |  |
| Were strategies to deal with confounding factors stated? | x |  |  |  | x |  |  |  | x |  |  |  |  | x |  |  |  | x |  |  |  | x |  |  |  | x |  |  | x |  |  |  |
| Were the outcomes measured in a valid and reliable way? | x |  |  |  | x |  |  |  | x |  |  |  | x |  |  |  | x |  |  |  | x |  |  |  | x |  |  |  | x |  |  |  |
| Was appropriate statistical analysis used? | x |  |  |  | x |  |  |  | x |  |  |  |  | x |  |  | x |  |  |  | x |  |  |  | x |  |  |  | x |  |  |  |
| **Overall apprraisal** | **Include** | | | | **Include** | | | | **Include** | | | | **Exclude** | | | | **Include** | | | | **Include** | | | | **Exclude** | | | | **Include** | | | |

|  | **Cardona 2016** | | | | **Catalan 2010** | | | | **Cortes 2014** | | | | **Cruz 2003** | | | | **De la rosa 2017** | | | | **Garces 2010** | | | | **Garcia 2014** | | | | **Gomez 2020** | | | | **Grajales 2011** | | | | **Grajales 2015** | | | |
| --- | --- | --- | --- | --- | --- | --- | --- | --- | --- | --- | --- | --- | --- | --- | --- | --- | --- | --- | --- | --- | --- | --- | --- | --- | --- | --- | --- | --- | --- | --- | --- | --- | --- | --- | --- | --- | --- | --- | --- | --- |
| **Question** | **Yes** | **No** | **Unclear** | **Not applicable** | **Yes** | **No** | **Unclear** | **Not applicable** | **Yes** | **No** | **Unclear** | **Not applicable** | **Yes** | **No** | **Unclear** | **Not applicable** | **Yes** | **No** | **Unclear** | **Not applicable** | **Yes** | **No** | **Unclear** | **Not applicable** | **Yes** | **No** | **Unclear** | **Not applicable** | **Yes** | **No** | **Unclear** | **Not applicable** | **Yes** | **No** | **Unclear** | **Not applicable** | **Yes** | **No** | **Unclear** | **Not applicable** |
| Were the criteria for inclusion in the sample clearly defined? | x |  |  |  | x |  |  |  |  |  | x |  | x |  |  |  |  |  | x |  | x |  |  |  | x |  |  |  | x |  |  |  | x |  |  |  | x |  |  |  |
| Were the study subjects and the setting described in detail? | x |  |  |  |  |  | x |  |  |  | x |  | x |  |  |  |  |  | x |  | x |  |  |  | x |  |  |  | x |  |  |  | x |  |  |  | x |  |  |  |
| Was the exposure measured in a valid and reliable way? |  |  | x |  | x |  |  |  | x |  |  |  | x |  |  |  | x |  |  |  | x |  |  |  | x |  |  |  | x |  |  |  | x |  |  |  | x |  |  |  |
| Were objective, standard criteria used for measurement of the condition? | x |  |  |  | x |  |  |  | x |  |  |  | x |  |  |  | x |  |  |  | x |  |  |  | x |  |  |  | x |  |  |  | x |  |  |  | x |  |  |  |
| Were confounding factors identified? |  | x |  |  |  |  |  | x |  |  |  | x |  | x |  |  |  | x |  |  | x |  |  |  | x |  |  |  | x |  |  |  | x |  |  |  | x |  |  |  |
| Were strategies to deal with confounding factors stated? |  | x |  |  |  |  |  | x |  |  |  | x |  | x |  |  |  | x |  |  | x |  |  |  | x |  |  |  | x |  |  |  | x |  |  |  | x |  |  |  |
| Were the outcomes measured in a valid and reliable way? | x |  |  |  | x |  |  |  | x |  |  |  | x |  |  |  | x |  |  |  | x |  |  |  | x |  |  |  | x |  |  |  | x |  |  |  | x |  |  |  |
| Was appropriate statistical analysis used? | x |  |  |  | x |  |  |  | x |  |  |  | x |  |  |  | x |  |  |  | x |  |  |  | x |  |  |  | x |  |  |  | x |  |  |  | x |  |  |  |
| **Overall apprraisal** | **Exclude** | | | | **Include** | | | | **Exclude** | | | | **Include** | | | | **Exclude** | | | | **Include** | | | | **Include** | | | | **Include** | | | | **Include** | | | | **Include** | | | |

|  | **Hernandez 2019** | | | | **Hilarion 2019** | | | | **Lopera 2019** | | | | **Martinez 2019** | | | | **Mejia 2007** | | | | **Ortiz 2018** | | | | **Osorio 2015** | | | | **Pinilla 2011** | | | | **Pinzon 2019** | | | | **Rivillas 2020** | | | | **Rubio 2008** | | | |
| --- | --- | --- | --- | --- | --- | --- | --- | --- | --- | --- | --- | --- | --- | --- | --- | --- | --- | --- | --- | --- | --- | --- | --- | --- | --- | --- | --- | --- | --- | --- | --- | --- | --- | --- | --- | --- | --- | --- | --- | --- | --- | --- | --- | --- |
| **Question** | **Yes** | **No** | **Unclear** | **Not applicable** | **Yes** | **No** | **Unclear** | **Not applicable** | **Yes** | **No** | **Unclear** | **Not applicable** | **Yes** | **No** | **Unclear** | **Not applicable** | **Yes** | **No** | **Unclear** | **Not applicable** | **Yes** | **No** | **Unclear** | **Not applicable** | **Yes** | **No** | **Unclear** | **Not applicable** | **Yes** | **No** | **Unclear** | **Not applicable** | **Yes** | **No** | **Unclear** | **Not applicable** | **Yes** | **No** | **Unclear** | **Not applicable** | **Yes** | **No** | **Unclear** | **Not applicable** |
| Were the criteria for inclusion in the sample clearly defined? | x |  |  |  | x |  |  |  | x |  |  |  | x |  |  |  |  |  | x |  | x |  |  |  | x |  |  |  | x |  |  |  | x |  |  |  | x |  |  |  | x |  |  |  |
| Were the study subjects and the setting described in detail? |  |  | x |  | x |  |  |  | x |  |  |  | x |  |  |  |  |  | x |  | x |  |  |  |  |  | x |  | x |  |  |  | x |  |  |  | x |  |  |  | x |  |  |  |
| Was the exposure measured in a valid and reliable way? | x |  |  |  | x |  |  |  | x |  |  |  | x |  |  |  | x |  |  |  | x |  |  |  |  |  | x |  | x |  |  |  | x |  |  |  |  |  | x |  | x |  |  |  |
| Were objective, standard criteria used for measurement of the condition? | x |  |  |  | x |  |  |  | x |  |  |  | x |  |  |  | x |  |  |  | x |  |  |  | x |  |  |  | x |  |  |  | x |  |  |  | x |  |  |  | x |  |  |  |
| Were confounding factors identified? | x |  |  |  | x |  |  |  | x |  |  |  | x |  |  |  | x |  |  |  | x |  |  |  | x |  |  |  | x |  |  |  | x |  |  |  |  |  | x |  |  | x |  |  |
| Were strategies to deal with confounding factors stated? | x |  |  |  | x |  |  |  | x |  |  |  | x |  |  |  | x |  |  |  | x |  |  |  | x |  |  |  | x |  |  |  | x |  |  |  |  |  | x |  |  | x |  |  |
| Were the outcomes measured in a valid and reliable way? | x |  |  |  | x |  |  |  | x |  |  |  | x |  |  |  | x |  |  |  | x |  |  |  | x |  |  |  | x |  |  |  | x |  |  |  | x |  |  |  | x |  |  |  |
| Was appropriate statistical analysis used? | x |  |  |  | x |  |  |  | x |  |  |  | x |  |  |  | x |  |  |  | x |  |  |  | x |  |  |  | x |  |  |  | x |  |  |  | x |  |  |  | x |  |  |  |
| **Overall apprraisal** | **Include** | | | | **Include** | | | | **Include** | | | | **Include** | | | | **Exclude** | | | | **Include** | | | | **Exclude** | | | | **Include** | | | | **Include** | | | | **Exclude** | | | | **Include** | | | |

Cohort studies

| **Reviewer: Laura Mora Moreo** |  |  |  |  |  |  |  |  |  |  |  |  |  |  |  |  |  |  |  |  |  |  |  |  |  |  |  |  |
| --- | --- | --- | --- | --- | --- | --- | --- | --- | --- | --- | --- | --- | --- | --- | --- | --- | --- | --- | --- | --- | --- | --- | --- | --- | --- | --- | --- | --- |
| **Date: 22/12/2021** | **Author/ year: Barahona 2020** | | | | **Charry 2008** | | | | **Charry 2009** | | | | **Devries 2015** | | | | **Egurrola 2019** | | | | **Howeuling 2016** | | | | **Paniagua 2011** | | | |
| **Question** | **Yes** | **No** | **Unclear** | **Not applicable** | **Yes** | **No** | **Unclear** | **Not applicable** | **Yes** | **No** | **Unclear** | **Not applicable** | **Yes** | **No** | **Unclear** | **Not applicable** | **Yes** | **No** | **Unclear** | **Not applicable** | **Yes** | **No** | **Unclear** | **Not applicable** | **Yes** | **No** | **Unclear** | **Not applicable** |
| Were the two groups similar and recruited from the same population? | x |  |  |  | x |  |  |  | x |  |  |  | x |  |  |  | x |  |  |  | x |  |  |  | x |  |  |  |
| Were the exposures measured similarly to assign people to both exposed and unexposed groups? | x |  |  |  |  |  | x |  |  |  | x |  | x |  |  |  | x |  |  |  | x |  |  |  | x |  |  |  |
| Was the exposure measured in a valid and reliable way? | x |  |  |  |  |  | x |  |  |  | x |  | x |  |  |  | x |  |  |  | x |  |  |  | x |  |  |  |
| Were confounding factors identified? |  | x |  |  | x |  |  |  | x |  |  |  | x |  |  |  | x |  |  |  | x |  |  |  | x |  |  |  |
| Were strategies to deal with confounding factors stated? |  | x |  |  | x |  |  |  | x |  |  |  | x |  |  |  | x |  |  |  | x |  |  |  | x |  |  |  |
| Were the groups/participants free of the outcome at the start of the study (or at the moment of exposure)? |  |  | x |  | x |  |  |  | x |  |  |  | x |  |  |  | x |  |  |  | x |  |  |  | x |  |  |  |
| Were the outcomes measured in a valid and reliable way? | x |  |  |  | x |  |  |  | x |  |  |  | x |  |  |  | x |  |  |  | x |  |  |  | x |  |  |  |
| Was the follow up time reported and sufficient to be long enough for outcomes to occur? | x |  |  |  | x |  |  |  | x |  |  |  | x |  |  |  | x |  |  |  | x |  |  |  | x |  |  |  |
| Was follow up complete, and if not, were the reasons to loss to follow up described and explored? | x |  |  |  | x |  |  |  | x |  |  |  | x |  |  |  | x |  |  |  | x |  |  |  | x |  |  |  |
| Were strategies to address incomplete follow up utilized? |  | x |  |  |  |  | x |  |  |  | x |  | x |  |  |  |  | x |  |  |  | x |  |  | x |  |  |  |
| Was appropriate statistical analysis used? | x |  |  |  | x |  |  |  | x |  |  |  | x |  |  |  | x |  |  |  | x |  |  |  | x |  |  |  |
| **Overral appraisal** | **Exclude** | | | | **Exlcude** | | | | **Exlcude** | | | | **Include** | | | | **Include** | | | | **Include** | | | | **Include** | | | |
